# Robust Controlled Degradation of Enzyme Loaded PCL‐Based Fibrous Scaffolds Toward Scarless Skin Tissue Regeneration

**DOI:** 10.1002/advs.202501053

**Published:** 2025-04-03

**Authors:** Lingling Fan, Weiliang Dong, Jianqi Lu, Yujia Peng, Bin Xie, Ping Wei, Min Jiang, Su Chen

**Affiliations:** ^1^ Key Laboratory for Waste Plastics Biocatalytic Degradation and Recycling College of Biotechnology and Pharmaceutical Engineering Nanjing Tech University Nanjing 211816 China; ^2^ State Key Laboratory of Materials‐Oriented Chemical Engineering Nanjing Tech University Nanjing 211816 China; ^3^ College of Chemical Engineering Nanjing Tech University Nanjing 211816 China

**Keywords:** degradation‐control fibrous scaffolds, enzyme‐loaded wound dressings, microplastics, non‐toxic by‐products, scarless skin tissue regeneration

## Abstract

Uncontrolled degradation of wound dressings may result in residues, causing several negative effects on wound healing, such as secondary damage, undesirable inflammation, and scar skin formation. Here, an available strategy associated with the synthesis of enzyme‐loaded (*Burkholderia cepacia* lipase, BCL) polycaprolactone (PCL) nanofiber scaffolds, aligning with wound healing effects is reported. These scaffolds are fabricated via fiber microfluidic electrospinning degradation‐control technique. The obtained scaffolds exhibit tunable degradation rates, achieving complete degradation within 12–72‐h cycles. The acidic degradation products are further elucidated and reveal the potential degradation mechanism. The acidic degradation products create an optimal microenvironment during the hemostasis and inflammation stages of wound healing. Notably, in vivo experiments demonstrate the enzyme‐loaded scaffolds effectively promote angiogenesis, reduce inflammatory responses, mitigate collagen deposition, and regulate fibroblast differentiation. This promotes rapid wound healing with a remarkable scarless rate of over 99% by day 21. New guidelines for scar‐free healing dressings are proposed, which carry out faster degradation without microplastics (MPs) and toxic byproducts before scar formation. These principles might provide valuable insights and promise for developing more effective wound dressings.

## Graphical abstract

A novel biodegradable wound dressing (E‐PCLs) based on polycaprolactone (PCL) and an enzyme complex (RHP‐BCL) is developed using a fiber microfluidic electrospinning technique. The dressing achieves complete degradation within 14 days, eliminates microplastic residues, and promotes scar‐free healing (99% in 21 days). This innovation provides a promising strategy for advanced wound care with synchronized degradation and healing.

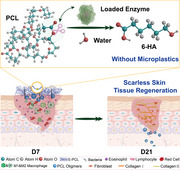

## 1 Introduction

From both fundamental and applied medical perspectives, the ability to perform wound dressings that promote scar‐free healing and prevent inflammatory responses is highly desirable. If performed in a controllable and reliable manner, such methods would benefit various research and development achievements by providing controllable and high‐efficiency biodegradable materials, while small molecules after degradation without toxic and adverse inflammatory reaction is a concern.^[^ ^1^ ^]^ Up to date, several spinning fiber biodegradable materials, such as polylactic acid (PLA), PCL, polylactide‐polyglycolide, and polyvinyl alcohol, are often applied to promote healing, reduce infection risk, minimize trauma, and support tissue regeneration as wound dressings.^[^ ^2^ ^]^ However, the degradation of materials is influenced by various factors.^[^ ^3^ ^]^ PCL and PLA, star medical materials, normally take over two years to fully degrade in the body.^[^ ^4^ ^]^ If MPs and small toxic molecules from their degradation process exist, it may lead to scar formation, inflammation, and other undesirable phenomena. On a more fundamental level, methods for enzyme‐loaded degradable biomaterials are available.^[^ ^5^ ^]^ However, there are few studies on the effect of in‐situ enzyme‐loaded biodegradable fibrous scaffolds on wound healing.

Enzymes, the products of nature's exquisite craftsmanship, are of versatility and unparalleled characteristics,^[^ ^6^ ^]^ such as highly catalytic activity, substrate specificity, and selectivity. The use of enzyme‐loaded biodegrade polymers allows them to accelerate controlled degradation fashion (days), while wound dressings confer scar‐free, avoiding secondary surgery^[^ ^7^ ^]^ and highly efficient healing.^[^ ^8^ ^]^ On the contrary, if wound dressings were employed with uncontrolled biodegradation of materials, they usually lead to significant negative effects, such as inflammation,^[^ ^9^ ^]^ scar formation,^[^ ^10^ ^]^ and other complications,^[^ ^11^ ^]^ and even cause lifelong issues for patients.^[^ ^12^ ^]^ Although the on‐demand dissolved hydrogels^[^ ^13^ ^]^ were activated by thermal, light, photothermal, ions and other solvents to be removed for several hours, the additional degradation conditions and potential remaining complex (such as poly (ethylene glycol) ‐thiols, aldehyde‐modified cellulose nanocrystals, aliphatic cyclic carbonate‐poly (ethylene glycol) and acetic acid) are still inconvenient for patients.^[^ ^14^ ^]^ Protein‐based wound dressings could be absorbed by body,^[^ ^15^ ^]^ while the nanozyme‐based^[^ ^16^ ^]^ or enzyme‐based^[^ ^17^ ^]^ wound dressings are employed to remove excess free radicals or necrotic tissue.

The bottom‐up approach is well illustrated by enzyme‐loaded biodegrade polymers, Gross et al.^[^ ^5a^ ^]^ initially utilized the anionic surfactant to protect *Candida antarctica* Lipase B to construct fast‐degradation cast films. The modified PCL‐based film embedded with 1.6% enzyme composite reached ≈90% weight loss in 9 days at 37°C in buffer solution. Iwata et al.^[^ ^17^ ^]^ developed self‐degrading aliphatic polyesters by embedding lipases through melt extrusion. All of them exhibited significant degradation within a short time frame. Furthermore, Marty et al.^[^ ^18^ ^]^ reported PLA‐based film loaded with 0.02 wt.% engineered enzyme (ProteinT*^FLTIER^*) could be decomposed within 20–24 weeks under home‐compost conditions. Xu et al.^[^ ^5c^ ^]^ employed the random heteropolymer (RHP) including hydrophobic and hydrophilic ends, which were designed and synthesized to realize the nearly complete degradation of PCL and PLA. Despite the indisputable achievements, most efforts seem to be far from easy to implement, and the attendant cost of preparation remains challenging.

**Figure 1:**
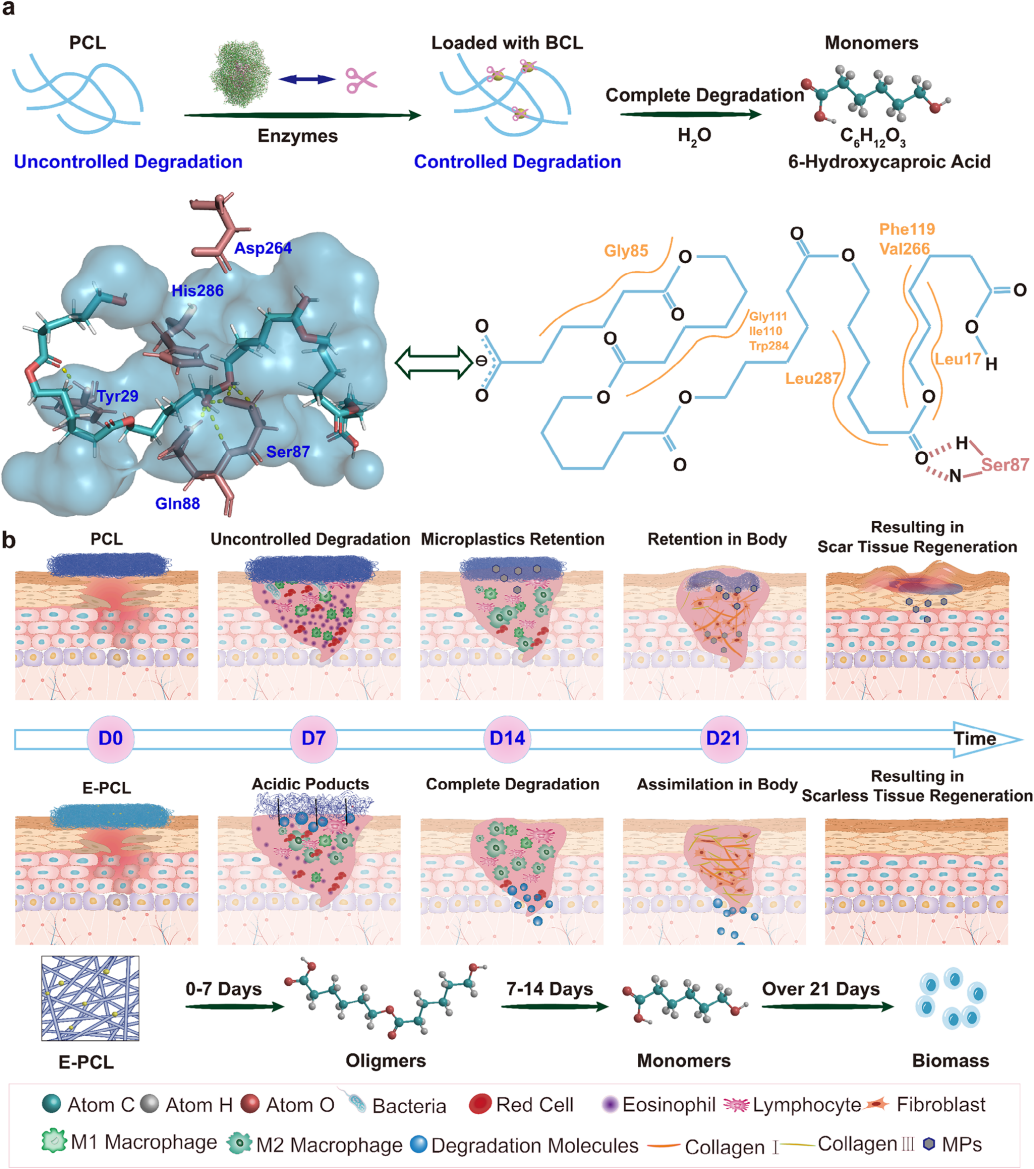
a) The strategy of self‐degrading scaffold construction and the reaction sites of PCL by BCL. b) The skin healing process associated with PCL and E‐PCL, as well as the degradation changes with E‐PCL.

We introduce a straightforward and effective method for producing self‐degrading wound dressings. These scaffolds loaded with enzymes (Abbreviated as E‐PCLs) can confer scarless skin regeneration by aligning with the wound healing process. We select BCL as the catalyst for PCL degradation and apply a microfluidic electrospinning degradation‐control technique to fabricate E‐PCLs. **Figure** **1**a shows the PCL loaded with enzyme can be decomposed into oligomers within 14 days. The enzyme protected by RHP may programmatically degrade PCL into 6‐hydroxycaproic acid (6‐HA) with size and space confinement effects. Thus, the skin wounds can mitigate the harmful effects of MPs. Perhaps more interesting, we confirm degradation products are non‐toxic and bioactive. They may maintain an optimal wound environment to promote skin tissue regeneration. Further illustrated in Figure 1b, the wound healing process contains four stages including hemostasis, inflammation, proliferation, and remodeling. The fully degradable E‐PCLs within 14 days play crucial roles in reducing inflammation, supporting cell proliferation, and promoting scar‐free tissue regeneration. Particularly, E‐PCL with 1 wt.% enzyme demonstrates nearly complete wound closure (98.68%) on day 14 and a 99.02% scarless skin formation rate on day 21. Ultimately, the as‐prepared self‐degrading wound dressing would be reached to minimize scar formation. This approach offers a practical strategy for wound care and inspires new materials for scarless skin healing.

## 2 Results and Discussion

### 2.1 Fabrication and Degradation‐Control Characterization of E‐PCLs

**Figure 2:**
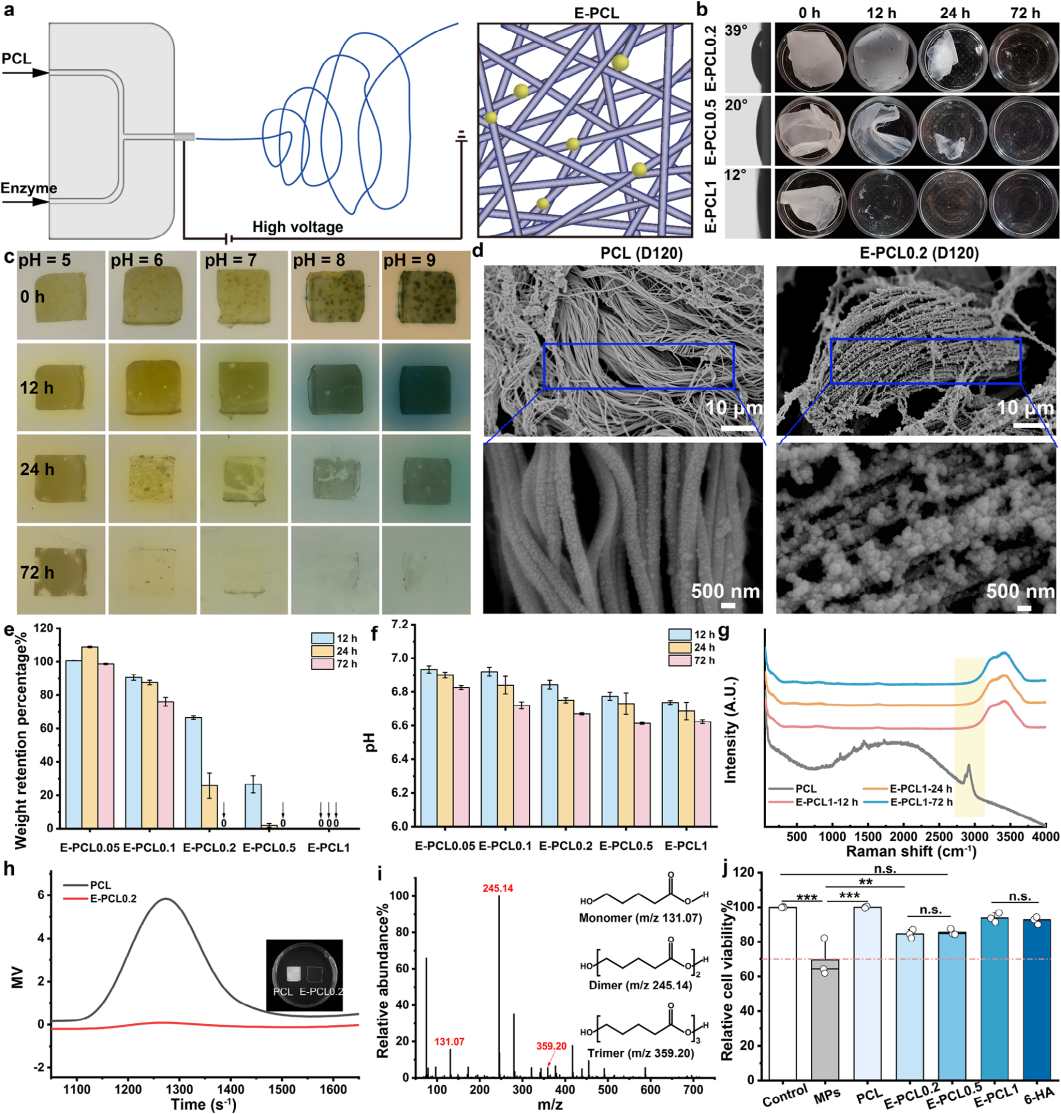
a) The fabrication process of E‐PCLs. b) Images of the water contact angle and degradation process of E‐PCLs in PB. c) Images of the degradation process of E‐PCL0.2 on the simulated wound surface with different pH values. d) Scanning electron microscope (SEM) images of PCL and E‐PCL0.2 on day 120 in subcutaneous implantation. e,f) Weight retention percentage and pH results of all groups. g) Raman spectra of original PCL fiber and degradation remaining of E‐PCL1. h) GPC results of PCL (the left) and E‐PCL0.2 (the right) on the simulated wound surface on hour 72. i) MS data of E‐PCL1 degradation solution on hour 72. j) Relative cell viability results of MPs, 6‐HA, and by‐products of PCL and E‐PCLs (100 µg mL^−1^) on hour 72.

We present here an easy‐to‐perform and versatile method enabling wound healing before fiber scaffold degradation. Initially, we fabricate scaffolds with varying enzyme concentrations via fiber microfluidic electrospinning degradation‐control technique (**Figure** **2**a). These scaffolds are loaded with BCL at concentrations of 0.05 wt.% (Abbreviated as E‐PCL0.05), 0.1 wt.% (Abbreviated as E‐PCL0.1), 0.2 wt.% (Abbreviated as E‐PCL0.2), 0.5 wt.% (Abbreviated as E‐PCL0.5), and 1 wt.% (Abbreviated as E‐PCL1). The enzymes are protected by RHP within the microfluidic electrospinning solution. As illustrated in Figure S1 (Supporting Information), the enzyme composites are circular and the mean diameter is ≈97.46 nm (PDI 0.13) in 2,2,2‐trifluoroethanol (TFE). By this protection, the enzyme activity of E‐PCL0.2 is over 27 times higher than that of PCL‐BCL (Figure S2, Supporting Information). In general, the improvement of hydrophilicity is beneficial to enhance the catalytic environment of enzymes. Figure 2b and Figure S3 (Supporting Information) demonstrate the reduction in water contact angle of E‐PCLs from 122.22 ± 2.12° to 13.59 ± 2.13° as the enzyme content increases. It may be ascribed to the structure of RHP with both hydrophilic and hydrophobic groups. Figure S4 (Supporting Information) shows RHP significantly reduces the water surface tension from 73.69 ± 0.03 to 44.13 ± 0.28 mN m^−1^. The RHP exhibits a surfactant‐like property with a critical concentration of 0.46 mg mL^−1^. Therefore, E‐PCLs may enhance the catalytic activity by accelerating water absorption. As illustrated in Figure 2b and Figure S5 (Supporting Information), all samples loaded with enzymes exhibit self‐degrading in phosphate buffer (PB). Especially for E‐PCL0.2, E‐PCL0.5, and E‐PCL1, they show nearly complete degradation within 72, 24, and 12 h, respectively. As shown in Figure S6 (Supporting Information), we further examine the microstructural changes of these samples. The PCL and PCL‐RHP scaffolds maintain intact structures, while the PCL‐BCL and E‐PCLs exhibit fiber fractures. Figure S7 (Supporting Information) illustrates PCL‐BCL degrades primarily through bulk degradation, whereas the E‐PCL0.05 sample undergoes surface erosion localized around the fibers. This degradation behavior may be beneficial for supporting cells during wound healing process. In addition, Figure S8 (Supporting Information) shows E‐PCLs have bead‐on‐string heterostructured nanofibers. The heterostructures can effectively encapsulate the enzyme composites, providing a controlled release. The enzyme composites serve as the hydrophilic surface and PCL as the hydrophobic surface. Due to their alternating hydrophilic and hydrophobic segments, these fibers are sensitive to relative humidity.^[^ ^19^ ^]^ As a result, the enzyme composites can partially swell and act as a microreactor, degrading the surrounding PCL segments. Therefore, the E‐PCLs with their excellent hydrophilicity and beaded structure demonstrate effective degradation in PB.

The second set of experiments focuses on the impact of pH value variation on wound surfaces. As depicted in Figure 2c, the E‐PCL0.2 exhibits excellent degradation effects on the simulated wound surface. It achieves nearly complete degradation within 72 h with pH values ranging from 7 to 9. In contrast, the degradation rates decrease at pH levels between 4 and 6. Therefore, E‐PCLs are unlikely to disrupt the normal skin's acid‐base balance. Next, Figure S9 (Supporting Information) demonstrates E‐PCL0.2 and its surrounding environment become acidic during degradation. The E‐PCLs may help mitigate the environmental pH values to normal skin levels. In this respect, Sharpe et al.^[^ ^21^ ^]^ concluded that differentiated keratinocyte phenotypes are promoted at lower pH levels. Kaufman et al.^[^ ^20^ ^]^ demonstrated that the epithelialization rate of guinea pig deep second‐degree burn wounds treated with a pH 3.5 solution was significantly higher (*p* < 0.001). These findings indicate an acidic microenvironment may promote wound healing.

Figure 2d shows the subcutaneously implanted E‐PCL0.2 underwent significant degradation after 120 days, whereas the PCL fiber structure shows no noticeable change. According to this literature,^[^ ^21^ ^]^ the degradation of PCL in the body primarily occurs through non‐enzymatic hydrolysis. Therefore, the addition of BCL significantly enhances the degradation rates of PCL, which is crucial for shortening foreign body reactions. Furthermore, we analyze the weight retention percentage and pH values during the E‐PCLs’ degradation process. As demonstrated in Figure 2e, the E‐PCL0.2, E‐PC0.5, and E‐PCL1 exhibit weight retention percentages of 67.47 ± 2.40%, 26.59 ± 5.17%, and 0% on hour 12, respectively. Additionally, the pH values of these samples exhibit a decreasing trend corresponding to weight changes in the E‐PCLs (Figure 2f). This is consistent with the results in Figure S9 (Supporting Information), confirming the generation of acidic byproducts. A few degradable fibrous wound dressings have been developed. A chitosan‐based fiber dressing only has a degradation rate of ≈28% after 28 days,^[^ ^22^ ^]^ However, complete re‐epithelialization occurs within 14–28 days.^[^ ^23^ ^]^ The E‐PCL0.2, E‐PCL0.5, and E‐PCL1 may meet this requirement.

### 2.2 Structural and Toxic Analysis of by‐Products

Another indication is that MPs (with a diameter smaller than 5 mm) highly impact the healing process. For example, it can harm the body and cells while triggering inflammatory and immune responses.^[^ ^9^, ^24^ ^]^ As indicated in Figure 2g, the degradation residues of E‐PCL1 have no PCL characteristic peaks at 2800–3200 cm^−1^ of C‐H stretching. This finding indicates MPs are hardly produced during the degradation process. We further detect the molecular distribution of all samples. As illustrated in Figure S10 (Supporting Information), samples such as E‐PCL0.2, E‐PCL0.5, and E‐PCL1 have no high‐molecular‐weight peaks (1000–1500 s⁻¹) in gel permeation chromatography (GPC) data after 72 h. In addition, Figure 2h exhibits the self‐degrading E‐PCL0.2 has no peak in the high‐molecular‐weight range on the simulated wound surface. These findings indicate E‐PCLs can degrade into low‐molecular‐weight fragments (less than 2000) under both degradation conditions. E‐PCLs may effectively reduce the issue of microplastic residues caused by the uncontrolled degradation of dressings.

The oligomers produced during degradation are usually not paid attention in previous studies.^[^ ^25^ ^]^ Figure 2i presents the mass spectrometry (MS) results of E‐PCL1 degradation solution at hour 72. The data reveals the presence of the monomer (6‐hydroxycaproic acid, 6‐HA, m/z = 131.07), the dimer (6‐((6‐hydroxyhexanoyl)oxy)hexanoic acid, m/z = 245.14), and the trimer (6‐((6‐((6‐hydroxyhexanoyl)oxy)hexanoyl)oxy) hexanoic acid, m/z = 359.20). Subsequently, Figure S11 (Supporting Information) shows the PCL is ultimately decomposed into 6‐HA after 96 h. The 6‐HA is an intermediate in fatty acid metabolism, involving redox reactions, β‐oxidation, and the citric acid cycle, which together produce energy in the body.^[^ ^26^ ^]^ Therefore, the programmable degradation of PCL is beneficial for wound healing. In vivo, natural enzymes typically anchor to the cell membrane or confine to specific micro‐ or nanoscale regions. This material mimics such an environment, providing an artificially confined bioreactor, and then replicating in vivo conditions.^[^ ^27^ ^]^ To further illustrate the potential degradation mechanism, we conduct molecular dynamics simulations and molecular docking. As depicted in Figure 1a and Figure S12 (Supporting Information), the ping‐pong enzymatic mechanism of BCL in degrading PCL involves a nucleophilic attack by Ser87. This reaction results in the departure of the alcoholic group on the ester substrate, ultimately leading to the cleavage of ester bonds. This process is facilitated by a catalytic triad (Ser87, His286, Asp264). Other amino acids can stabilize the PCL structure (e.g. Gln88 and Tyr29), enabling efficient substrate recognition, binding, and subsequent bimolecular nucleophilic substitution (S*_N_* 2) reactions.^[^ ^28^ ^]^ The programmable degradation results are consistent with the mechanism outlined in a previous study by Xu and coworkers.^[^ ^5c^ ^]^ However, unlike the research in molecular dynamics simulations, Figure S13 (Supporting Information) exhibits the RHP does not disperse in water, even at 400 K for 40 ns and 310 K for 200 ns. Notably, the reactive pocket remains exposed at specific times, creating a “narrow channel” for the entrance of PCL. Enzyme composites are confined in fiber, further limiting the dissociation of RHP. These spatial constraints facilitate a more precise and controlled degradation of PCL, enhancing the effectiveness of the degradation process. The enzyme bioreactor allows PCL linear molecules to interact efficiently with the active sites of BCL. This interaction facilitates the programmed degradation of PCL.

Figure 2j and Figure S14 (Supporting Information) display the cell toxicity of degradation products. According to the ISO 10993–5:2009 standard,^[^ ^29^ ^]^ the results further indicate that all tested E‐PCLs maintain a non‐toxic status (> 70%) at a concentration of 100 µg mL^−1^. Notably, 6‐HA exhibits favorable relative cell viability (89.55 ± 1.13%) even at 200 µg mL^−1^, confirmed by the previous study on human fibroblasts, up to 2643.2 µg mL^−1^.^[^ ^30^ ^]^ Additionally, E‐PCL1 (94.01 ± 2.65%) demonstrates superior relative cell viability compared with E‐PCL0.2 (84.54 ± 2.41%) and E‐PCL0.5 (85.52 ± 1.70%), indicating low acute toxicity and good biocompatibility. However, the MPs demonstrate a relative cell viability of 69.51 ± 11.04% and significantly inhibit cell growth. Studies show that MPs can activate the ROS/NLRP3/Caspase‐1 pathway, causing intestinal inflammation.^[^ ^22^ ^]^ In contrast to hydrogels with rapid degradation rates, E‐PCLs distinctly produce simple, non‐toxic degradation products, a crucial characteristic for biomedical applications. For instance, although aloe vera‐based hydrogels are green and biodegradable, the complex nature of their degradation products continues to pose significant safety concerns.^[^ ^31^ ^]^

### 2.3 Assessment of Stability, Mechanical Strength, and Gas Permeability in E‐PCLs

We further investigate the stability tests on E‐PCLs structures. Figure S15 (Supporting Information) exhibits the E‐PCL0.2 has no significant degradation of fibers at 37°C in air after 3 months. The E‐PCLs display excellent storage stability. Subsequently, we examine the degradation behavior of E‐PCLs after 1 year of storage at room temperature. Figure S16 (Supporting Information) illustrates E‐PCL1 achieves nearly complete degradation within 12 h in PB, while E‐PCL0.2 and E‐PCL0.5 also demonstrate notable degradation performance. As shown in Figure S17 (Supporting Information), the degradation rate of E‐PCL0.2 and E‐PCL0.5 slows down, with the weight retention percentage increasing by ≈10% after 12 h. Nevertheless, they can still cause changes in pH values. Furthermore, the activities of E‐PCL0.2, E‐PCL0.5, and E‐PCL1 retain 52.51 ± 4.62%, 61.93 ± 33.37%, and 81.83 ± 3.86% of their initial levels, respectively. The E‐PCLs demonstrate a swift degradation rate and exceptional long‐term stability. These results collectively evidenced this approach offers a more versatile solution to regulate degradation rates and reduce MPs retention. More importantly, the E‐PCLs may undergo self‐degradation in alignment with the wound‐healing process. This allows the strategy to be more efficient, safer, and eco‐friendly.

The following experiment is focused on the properties of wound dressings, including mechanical properties and gas permeability. As shown in Figure S18 (Supporting Information), E‐PCL0.2 exhibits superior elongation at break and tenacity compared to PCL, indicating enhanced plasticity and impact resistance in E‐PCLs formulations. Notably, the tenacity and Young's modulus of E‐PCL0.2 is recorded at 1253.24 MPa·m^1/2^ and 46.61 kPa respectively, which is significantly higher than those of the previous study.^[^ ^32^ ^]^ As E‐PCLs degrade, their mechanical tension decreases significantly, potentially promoting scarless tissue regeneration by effectively shielding against tension. Adequate gas permeability is crucial for wound dressings to effectively regulate heat and humidity. Despite the bead‐like structures of E‐PCL0.2, it maintains a uniform pore size distribution with an average pore size of 2.08 µm. The E‐PCL0.2 also achieves a high permeability of 834.40 ± 0.03 m^3^/(m^2^·h·kPa) (Figure S19, Supporting Information). It significantly surpasses the nitrogen transmissibility (164.63 m^3^/(m^2^·h·kPa)) of the reported sealant‐loaded nanofiber biomimetic scaffold of artificial skin.^[^ ^5c^ ^]^ As E‐PCLs degrade, their gas permeability increases, reducing the risk of exogenous infection and enhancing the comfort of the injured area. These findings confirm E‐PCLs have excellent mechanical properties and gas permeability. E‐PCLs, with these advantageous properties, show good potential as an effective wound dressing.

### 2.4 In Vivo Efficacy of Wound Healing Using E‐PCLs

**Figure 3:**
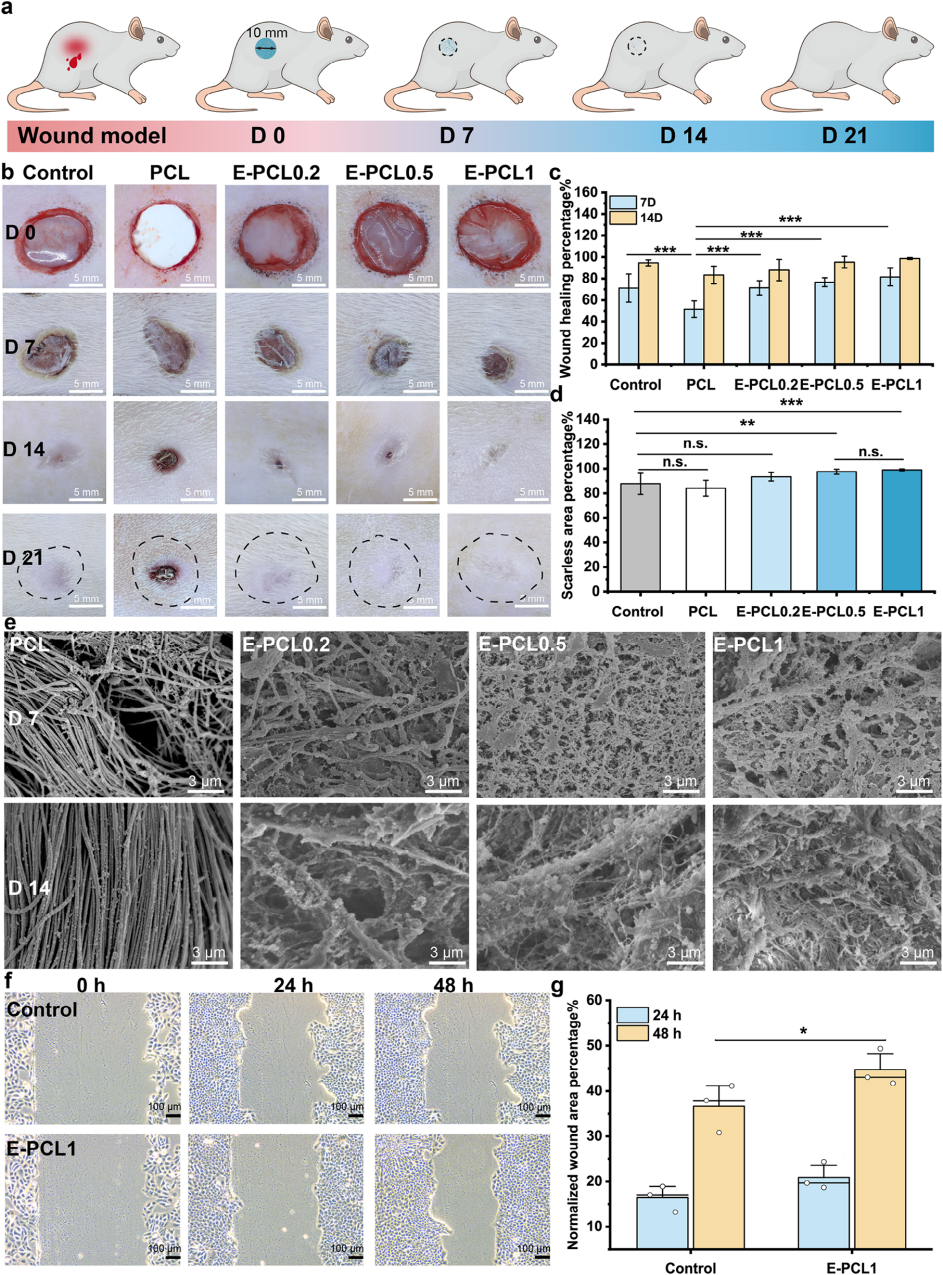
a) Schematic illustration of E‐PCL process for scarless skin formation. b) Photos of the wound area after the rat received different treatments (*n* = 6 biologically independent samples). c) Graph of the wound healing percentage (on days 7 and 14) and d) scarless area percentage (on day 21) of the Control, PCL, and E‐PCLs. e) SEM images of the wound surface on day 7 and 14, respectively. f) HaCaT cell migration images and g) data of Control and byproducts of E‐PCL1.

To test the efficacy of E‐PCLs in healing wounds, a full‐thickness excisional defect mouse model is established. **Figure** **3**a illustrates E‐PCLs can degrade as wound healing. Figures S20 and S21 (Supporting Information) show E‐PCL0.2 demonstrates a hemostatic effect within ≈20 s and prevents significant blood loss. As shown in Figure 3b, wounds treated with E‐PCLs demonstrate superior performance. Figure 3c shows E‐PCL0.2 achieves a 71.36 ± 6.60% healing efficiency and E‐PCL0.5 reaches 76.59 ± 4.12%. Particularly, E‐PCL1 exhibits over 81% healing efficiency, in contrast to 51.48 ± 7.68% for the PCL and 71.19 ± 13.08% for the negative control groups. By day 14, all E‐PCL treated groups display nearly complete wound closure, with healing efficiencies of 87.86 ± 9.88%, 95.25 ± 5.41%, and 98.68 ± 0.84%, respectively. As shown in Figure 3d, the PCL reaches a scarless area percentage of 87.74 ± 8.62% and the control group achieves 84.10 ± 6.42%. Notably, the E‐PCL0.2, E‐PCL0.5, and E‐PCL1 groups show minimal scar formation by day 21, achieving 93.41 ± 3.45%, 97.50 ± 2.03%, and 99.02 ± 0.76% scarless skin area percentage, respectively. The potential factor enabling E‐PCLs to promote scarless skin formation is their degradation capability. Such as polyesters‐based triols (900 g mol^−1^), the residues impede wound healing and lead to scar formation.^[^ ^33^ ^]^ Extra extracellular matrix (ECM) may lead to scar formation.^[^ ^9a^ ^]^ As illustrated in Figure 3e, compared with PCL groups, all groups treated with E‐PCLs exhibit markedly greater degradation after 7 days of incubation. This degradation becomes increasingly pronounced in the E‐PCL0.2, E‐PCL0.5, and E‐PCL1 groups. By the time the wound has nearly closed at 14 days, E‐PCL0.2 still retains its fibrous structure, while E‐PCL0.5 and E‐PCL1 have been completely degraded. For PCL, the delayed degradation of the wound dressings potentially contributes to foreign body reactions and excessive deposition. The unsuitable degradation rate may lead to scar formation. We further evaluate the impact of degradation products on cellular processes to better demonstrate their role in enhancing wound closure. As shown in Figure 3f,g, the degradation products (a mixture of acidic oligomers) of E‐PCLs promote cell migration after 48 h with a 44.69 ± 4.10% scratch healing rate. Keratinocyte migration has been shown to increase antioxidant enzyme levels, including superoxide dismutase (SOD).^[^ ^34^ ^]^ However, under oxidative stress, HaCaT cell‐to‐cell junctions are inhibited.^[^ ^35^ ^]^ Figure S22a (Supporting Information) shows the 1,1‐diphenyl‐2‐picrylhydrazyl (DPPH) scavenging rates of 6‐HA and E‐PCL at 17.79 ± 4.25% and 9.11 ± 1.57%, respectively, aligning with literature results.^[^ ^36^ ^]^ Previous studies reported that 6‐HA exhibited antioxidant properties due to its hydroxyl groups, which donated hydrogen and electrons to free radicals.^[^ ^37^ ^]^ Furthermore, Figure S22b (Supporting Information) displays E‐PCL1 (100 µg mL^−1^) significantly enhanced SOD activity in the protection group, reducing oxidative damage and ultimately promoting HaCaT cell migration, even promote wound closure. Therefore, E‐PCLs offer great wound protection, support cell proliferation, and may help restore the normal skin microenvironment, which may further promote wound healing.^[^ ^14^ ^]^ These findings affirm the potential of E‐PCL0.5 and E‐PCL1 with their suitable degradation rates, minimizing damage from foreign materials to the wound, and reducing the formation of heterogeneous structures.

### 2.5 Impacts of E‐PCL on Inflammation and Collagen During Wound Healing

**Figure 4:**
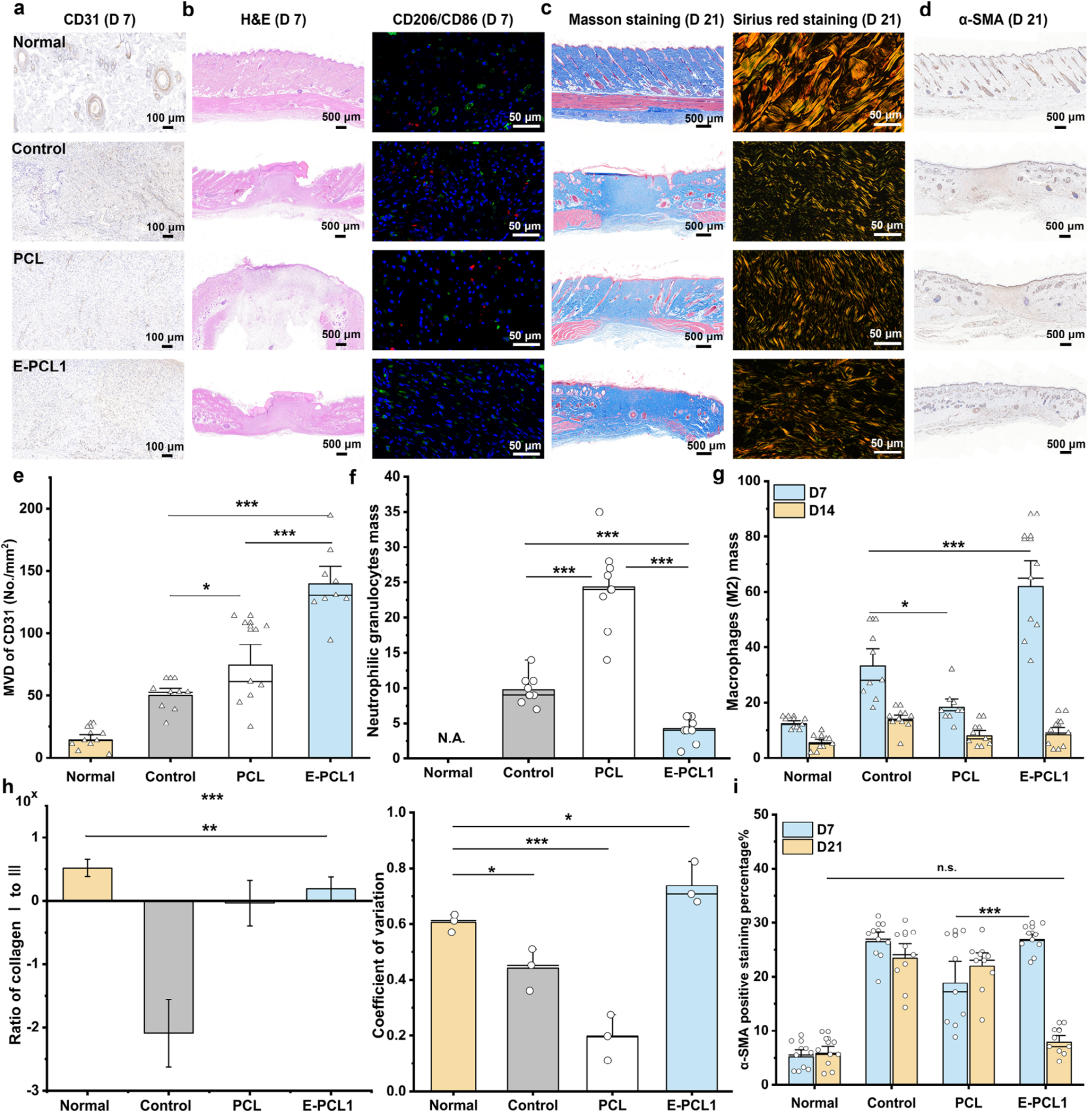
a) Images of the CD31 staining on day 7. b) Images of the H&E staining and CD86/CD206 immunohistochemical staining on day 7, respectively. c) Images of the Masson staining, Sirius red staining, and α‐SMA staining on day 21. d) Images of the α‐SMA staining on day 21. e) The microvascular density (MVD) of CD31. f) Quantitative analysis of neutrophilic granulocytes. g) Quantitative analysis of macrophages (M2). h) Quantitative analysis of the collagen distribution and collagen fiber angle variability coefficient on day 21. i) The α‐SMA positive staining percentage of all groups on days 7 and 21. The study includes three biologically independent samples (*n* = 3).

As illustrated in **Figure** **4**, we further assess the ability of E‐PCL1 to promote angiogenesis, reduce inflammatory responses, mitigate collagen deposition, and regulate fibroblast differentiation in vivo. Figure 4a shows E‐PCL1 exhibits a higher number of platelet‐endothelial cell adhesion molecule‐1 (CD31)‐positive dots compared to PCL, indicating increased microvessel formation. Figure 4b and Figures S23 and S24 (Supporting Information) display the Control and PCL groups exhibit larger areas densely populated with inflammatory cells (e.g. neutrophils and macrophages) as observed in hematoxylin‐eosin staining and CD86/CD206 immunohistochemical staining. The E‐PCL1 group significantly reduces the inflammation in wounds. As shown in the Masson staining results (Figure 4c; Figure S25, Supporting Information), the E‐PCL1 group exhibits higher collagen deposition (blue area) than that of the PCL and control groups on day 21. Additionally, Figure S26 (Supporting Information) illustrates the epithelial thickness in E‐PCL1‐treated wounds appears more uniform. This observation suggests the potential for scarless skin formation. The Sirius red staining data further demonstrates collagen arrangement in the E‐PCL1 group more closely resembles that of normal skin. Figure 4d and Figure S27 (Supporting Information) display E‐PCL1 exhibits a larger α‐smooth muscle actin (α‐SMA)‐positive area than PCL on day 7, but a significantly lower area than the Control and PCL groups on day 21. This indicates E‐PCL1 promotes early angiogenesis while preventing excessive fibrosis in the later stage, supporting scarless wound healing.^[^ ^38^ ^]^

To further confirm the efficacy of E‐PCL1, we analyze tissue staining results. As shown in Figure 4e, E‐PCL1 exhibits significantly higher microvascular density (MVD) of CD31 compared to Control and PCL. CD31 serves as a marker of vascular endothelial differentiation, and its increased expression, along with the earlier formation of new blood vessels, indicates a faster wound‐healing process.^[^ ^39^ ^]^ Furthermore, Figure 4f exhibits the PCL group has more neutrophilic granulocyte cells than the E‐PCL1 group. Generally, the presence of neutrophils, which are closely associated with infection. As illustrated in Figure 3g, the acidic products generated from E‐PCLs’ self‐degrading occur within the first 7 days. Skin injury compromises its antibacterial function, primarily by disrupting the skin's naturally weak acidic barrier. The antibacterial efficacy of the scaffolds is tested on the plates against *Escherichia coli* (*E. coli*) and *Staphylococcus aureus* (*S. aureus*) (Figure S28, Supporting Information). The areas covered by E‐PCL0.2 and E‐PCL0.5 show no colony formation. However, E‐PCL1 shows negligible antibacterial activity against *E. coli*. These findings suggest the degradation products can reduce bacterial infection by inhibiting bacteria growth. Additionally, M1 macrophages primarily secrete pro‐inflammatory cytokines and chemokines, while M2 macrophages predominantly produce anti‐inflammatory cytokines. And M2 macrophages play a crucial role in wound healing and tissue repair.^[^ ^40^ ^]^ The immunohistochemical analysis using CD86 and CD206 markers demonstrates the E‐PCL1 treated wounds have a significant shift from M1 (red) to M2 (green) macrophages by day 7 (Figure 4g; Figure S29a, Supporting Information). By day 14, the number of macrophages returned to normal levels (Figure S29b, Supporting Information). These results indicate the degradation and acidic products produced by E‐PCL1 effectively inhibit bacterial infections and promote the transition to M2 macrophages. Therefore, E‐PCL1 reduces the inflammation to promote wound healing.

In addition, overproduction of ECM components including collagen can cause excessive fibrosis, which may lead to scar formation. Type III collagen contributes to the softness and elasticity of the skin, while type I collagen provides a structural framework essential for skin regeneration.^[^ ^41^ ^]^ Figure 4h reveals an excessive amount of type III collagen in the control group, indicating immature wound healing or potential scar formation. In contrast, the E‐PCL1 group shows a balanced ratio of type I to type III collagen. This finding indicates the potential for normal, scarless skin formation. Figure S30 (Supporting Information) demonstrates collagen fibers in the E‐PCL1 group are loosely arranged and lack organization. Hypertrophic scars feature elevated parallel‐aligned type III collagen, while keloid scars show excess disorganized types I and III collagen.^[^ ^42^ ^]^ These results indicate the E‐PCL1 treated wounds have appropriate collagen deposition and a favorable ratio of disorganized types I and III collagen.

Furthermore, fibroblast differentiation into α‐SMA‐positive myofibroblasts is another key factor associated with hypertrophic scar formation.^[^ ^38^ ^]^ As illustrated in Figure 4i, α‐SMA expression is high in wound groups overall, with the E‐PCL1 group displaying a significant increase in α‐SMA‐positive cells on day 7. Early on, α‐SMA serves as a marker of smooth muscle cell differentiation, indicating angiogenesis alongside CD31. However, as wound remodeling progresses and vascular demand decreases, α‐SMA expression may decline.^[^ ^43^ ^]^ Notably, while the Control and PCL groups maintain high α‐SMA levels, the E‐PCL1 group restores them to normal, suggesting its role in preventing excessive fibrosis to promote scarless wound healing. The observed promotion of angiogenesis, anti‐inflammatory properties, modulation of collagen deposition, and regulation of fibroblast differentiation highlight the potential of E‐PCLs. These characteristics suggest they can serve as promising candidates for advancing scarless wound healing therapies.

## 3 Conclusion

In this work, we have found an available strategy for scarless tissue regeneration for skin wounds. That is, we have developed enzyme‐loaded PCL nanofiber scaffolds via fiber microfluidic electrospinning degradation‐control technique toward robust wound healing. These scaffolds exhibit tunable degradation rates, achieving complete breakdown within 12, 24, and 72 h for 1, 0.5, and 0.2 wt.% enzyme‐loaded concentrations in vitro, respectively. The enzyme complex facilitates the programmable degradation of PCL via ester bond cleavage, leading to the degradation product of 6‐HA and other oligomers. All products were verified to be non‐toxic and possess antibacterial properties. This strategy is versatile, and various types of wound dressings could be used in this method. On one hand, these dressings create an optimal microenvironment of wound healing by mimicking the acidic conditions of normal skin, generating bioactive degradation products, and leaving no excess ECM‐like residues. On the other hand, the method rapidly allows PCL nanofiber scaffolds to completely degrade, along with no MPs, fostering the regeneration of scar‐free skin tissue. This microenvironment may further promote angiogenesis, reduce inflammatory responses, mitigate collagen deposition, and regulate fibroblast differentiation. In practice, we propose that the complete degradation within 14 days is crucial for preventing scar formation. Specifically, E‐PCL1 could degrade completely within 12 h in vitro and 14 days in vivo, and has shown remarkable efficacy: achieving 81% healing efficiency by day 7%, 99%, and 100% wound closure by days 14 and 21, correspondingly. The scarless skin formation is over 99% by day 21. The importance of complete dressing degradation before scar formation (ideally within 14 days), the presence of non‐toxic degradation products, and the elimination of MPs residues during the wound healing process. The controlled enzyme‐loaded self‐degrading wound dressings would benefit scarless tissue regeneration, therefore, offer valuable insights into regenerative medicine and clinical settings.

## 4 Experimental Section

### Materials

Amano Lipase PS, from *Burkholderia cepacia* (BCL, Mw 34.1 kDa) and polycaprolactone (PCL, average Mn 80 000) were purchased from Sigma–Aldrich. Methyl methacrylate (MMA, 99%, Aldrich), ethylene glycol methyl ether methacrylate (OEGMA, 99%, Mn = 500 g mol^−1^, Aldrich), and 2‐ethylhexyl methacrylate (2‐EHMA, 98%, Aldrich) were passed through a short column of neutral alumina to remove inhibitor before use. PB solution was prepared with 50 mm sodium dihydrogen phosphate and sodium dihydrogen phosphate adjusting its pH to 7.0. The TFE, 6‐HA Potassium 3‐(methacryloyloxy) (SPMA) with analytically pure were purchased from Shanghai Aladdin Reagent Co., Ltd. (Shanghai, China).

### Preparation and Characterization of RHP‐BCL

The RHP was prepared according to the previous reports.^[^ ^44^ ^]^ Briefly, the RHP was synthesized by mixing MMA (2 mmol), OEGMA (4 mmol), EHMA (2.5 mmol), and SPMA (0.50 mmol) with the ratio of 2: 4: 2.5: 0.5 (mol mol^−1^), azobisisobutyronitrile (AIBN, 0.06 mmol) and S‐methoxycarbonylphenylmethyl dithiobenzoate RAFT (0.04 mmol) and N, N‐Dimethylformamide (12 mL), then degassed by three freeze‐pump‐thaw cycles and stored at 60 °C for 24 h with nitrogen. It was then purified with water by dialysis membranes (5000 M mol^−1^) for 3 days. Finally, 2 g RHP was obtained by freeze‐drying the remaining solution. The ^1^H NMR and ^13^C NMR were carried out with Bruker 300 (DMSO‐*d6*, 300 MHz) of RHP. Surface tension measurements were conducted for RHP at various concentrations ranging from 0 to 0.8 mg mL^−1^ by automatic surface tensiometer (Shanghai Pingxuan Scientific Instrumnet Co., Ltd.). BCL was purified by using the desalting column (HiPrepTM 26/10) and concentrated by using an ultrafiltration spin column (15 mL, 10 kDa MWCO, PES, Sartorius Sub‐packaging). Bicinchoninic Acid Assay detected the concentration of the BCL with the BCA kit (Labgic Technology Co., Ltd).

To obtain the RHP‐BCL complex, RHP (Mn 19.30 kDa, 0.3 mm, aqueous solution) was added to the BCL aqueous solution at a ratio of 100/1 (mol mol^−1^), then incubated at 30°C with agitation at 200 rpm for 10 min. After that, the complex was prepared by freeze‐drying. The morphology of RHP‐BCL in TFE was assessed by SEM (ZEISS Sigma 300). The particle size was assessed by dynamic light scattering (DLS, Zetasizer Nano ZS90).

### Preparation of E‐PCLs by Fiber Spinning Degradation‐Control Technique

The scaffolds were manufactured by a microfluidic electrospinning device (Nanjing Janus New‐Materials Co. Ltd). PCL and enzyme composites were prepared in TFE and spun into fibers using a Y‐type coaxial microfluidic chip, with varying flow rates to control fiber composition. The PCL solution (13 wt.%) and additional composites (185 mg mL^−1^) were prepared using TFE. The well‐dispersed solutions were separately injected into the microchannel by syringes to form fibers. The flow rate of PCL solutions was fixed at 0.5 mL h^−1^. Meanwhile, the flow speed of enzyme composites was integrated with the requirements. The E‐PCL0.05, E‐PCL0.1, E‐PCL0.2, E‐PCL0.5, and E‐PCL1 with the flow speed at 0.01, 0.02, 0.04, 0.1, 0.2 mL h^−1^, respectively. The PCL‐RHP and PCL‐BCL have the same flow as the E‐PCL0.2. The resulting solution was spun for 5 h at 450 rpm under a voltage of 18 kV for collecting the fibers. The collected fibers were then dried at room temperature overnight in a vacuum oven to remove the residual TFE. The scaffolds with thymol blue (0.5 wt.%) were fabricated based on E‐PCL0.2%.

The enzyme activity of the scaffolds and enzyme composites was measured by the reaction with 4‐nitrophenyl hexanoate (pNP‐C6). The generation rate of *p*‐nitrophenol was recorded at the wavelength of 410 nm. One enzyme activity unit (U) of BCL was defined as the amount of enzyme required to hydrolyze pNP‐C6 producing 1 µmol *p*‐nitrophenol during 1 min at 37 °C. The surface wettability was assessed with a contact angle analyzer (DSA100, KRÜSS, Germany). The mechanical properties of the fibers were studied using an electronic universal testing machine (SANS). The pore size distribution and the gas (Nitrogen) permeability of the scaffolds were analyzed by Capillary Flow Porometer (PMI ipore1500).

### Characterization of E‐PCLs Degradation

The 6 mg scaffolds were introduced into the PB at a final concentration of 2 mg mL^−1^. The samples were degraded at 37°C. Taking photographs and samples on hours 0, 12, 24, and 72. The samples were dried in a vacuum chamber and weighed at each extraction time. The weight retention percentage of the samples was calculated using the following Equation (1):

(1) $$\Delta m\left(\%\right)=\frac{m_{t}}{m_{0}}\times 100\%$$

where *m_0_* is the initial mass of the scaffolds and *m_t_* is the mass of the dry scaffolds after a given time of immersion. The remaining degradation scaffolds were confirmed by SEM (ZEISS Sigma 300), ATR‐FTIR (Thermo Scientific™ Nicolet™ iS™), Raman spectra (Horiba LabRAM HR Evolution) and GPC (Waters 1515).

### Molecular Dynamics Simulation and Molecular Docking

The structure of BCL protected by RHP was obtained based on molecular dynamics simulation results in water as described in a previous study.^[^ ^44^, ^45^ ^]^ Specifically, the simulation was initially conducted under vacuum conditions, followed by simulations at 400 K for 40 ns to promote self‐assembly. This was succeeded by a production simulation at 310 K for 200 ns in water. The final frame from this simulation served as the input protein structure for subsequent molecular docking studies. A hexamer of PCL was employed as a representative structure for docking studies. To explore potential interactions, multiple conformers of PCL were generated using the Confab package from Open Babel.^[^ ^46^ ^]^ These conformers were subsequently docked into the active site of the receptor using a custom AutoDock,^[^ ^47^ ^]^ which allowed for flexibility in both the backbone and side chains during the docking simulations. The 2D view of docking results was analyzed by Proteins Plus Server (https://proteins.plus/). All molecular structures were visualized using PyMOL software (https://pymol.org/2/).

### Degradation Experiments on the Simulated Wound Surface

According to the study,^[^ ^48^ ^]^ the simulated wound surface was prepared with 1.2% (w/v) agar powder added to the simulated wound fluid. The composition of the simulated wound fluid is as follows: 29 g L^−1^ bovine serum albumin, 18.8 mmol L^−1^ ethyl lactate, 105 mmol L^−1^ creatinine, 2.5 mmol L^−1^ CaCl_2_, 142 mmol L^−1^ NaCl, 0.7 mmol L^−1^ glucose, and 9.6 mmol L^−1^ urea. The PCL and E‐PCLs were cut into rectangular strips with 2×2 cm and weighted by electronic balance. The plates were incubated at 37 °C. The remaining degradation scaffolds (PCL, E‐PCL) and the agar plates covered by them were dissolved in tetrahydrofuran for characterization by GPC.

### Cytotoxicity Tests of the Self‐Degrading Scaffolds

The degradation products of MPs (produced by crusher), PCL, E‐PCLs, and 6‐HA were sterilized by Ultraviolet irradiation sterilization. 4 × 10^4^ NCTC clone 929 cells (L929 fibroblasts) were seeded per well. The cell viability of experimental groups was measured with the cell counting kit‐8 test and expressed by the percentage of living cells concerning the control cells. The relative cell viability was calculated using the following Equation (2):

(2) $$\mathrm{Relative}\mathrm{cell}\mathrm{viability}\left(\%\right)=\frac{A_{i}-A_{0}}{A_{control}-A_{0}}\times 100\%$$

where *A_i_* is the intensity of the well added with samples at 450 nm, *A_0_* is the intensity of the well without anything added at 450 nm and *A_control_* is the intensity of the well without samples at 450 nm.

### Subdermal Implantation of the Self‐Degrading Scaffolds

According to ISO 10993‐6, four subcutaneous capsules with a depth of 10 mm from the skin incision were prepared on the back of each *Sprague Dawley* (SD) rat. All procedures were conducted in accordance with the “Guiding Principles in the Care and Use of Animals” (China) and were approved by The Animal Experimentation Ethics Committee of Research selection Biotechnology (Hangzhou) Co., LTD (YXSW2303301023). The control was implanted with PCL with the size of 10×10 mm and the experimental group was implanted with the same size of E‐PCL0.2. The experimental SD rats were dissected on day 120, and the subcutaneous tissue containing the material was immediately fixed in 2.5% glutaraldehyde in a 2 mL centrifuge tube and stored at 4°C overnight. After discarding the fixative, the sample was rinsed three times with 0.01 M phosphate‐buffered saline (PBS, pH 7.4) for 15 min each. The tissue was then post‐fixed with 1% osmium tetroxide for 1 h, followed by three additional PBS rinses. To dehydrate the sample, it was exposed to increasing ethanol concentrations (30%, 50%, 70%, and 90%) for 15 min each, followed by 100% ethanol for 20 min. The dehydrated samples were dried using a critical point dryer with specific settings, and then sputter‐coated with platinum for 70 s. Finally, the implanted tissue was taken SEM (ZEISS Sigma 300) to detect the morphology of the samples.

### Liver Hemorrhage Experiment

Female SD rats (6–8 weeks) were randomly divided into three groups. The rats were fixed on the surgical plate with tilted 45° (Approval Number: YXSW2303301023). A piece of liver tissue with a length of 1.5 cm and a width of 0.5 cm was cut off at the lower edge of the liver lobe. The wound was covered with PCL and E‐PCL0.2, respectively, and the wound without materials was covered as the control group. The bleeding liver hemostasis of each group of rats was recorded by video recording. The weight loss of the blood was calculated using the following Equation (3):

(3) $$m_{loss}=m_{t}-m_{0}$$

where *m_t_* is the mass of the mass of the filter paper and lost blood and *m_0_* is the initial mass of the filter paper.

### Antibacterial Activity Assays

The *E. coli* (ATCC 25922) and *S. aureus* (ATCC 6538) were cultured on Luria–Bertani medium overnight. The 2 × 2 cm scaffolds were introduced on the plates with 100 µL bacteria liquid at 37 °C for 12 h.

### Full‐Thickness Cutaneous Wound Healing

The rounded full‐thickness cutaneous wound area (the circle with a diameter of ≈10 mm) was created on the back of each SD rat to establish a wound model (Approval Number: YXSW2303301023). Wounds were used as control covered by nothing and PCL, respectively, and the other wound was used as an experimental group covered by E‐PCLs. During the period of wound healing, the materials were not removed. The wounds were recorded and the area was calculated by ImageJ. The tissue sections were stained with H&E on days 7 and 14, Masson's trichrome on day 21, and CD31 on day 7. Additionally, Sirius Red and α‐SMA staining were performed on days 7 and 21. Immunohistochemical staining was conducted using anti‐CD86 for M1 macrophages and anti‐CD206 for M2 macrophages. Quantitative analysis was performed on three regions of interest areas for each sample. To analyze the morphology of the samples by SEM on days 7 and 14, respectively. The wound and scar area are analyzed by ImageJ image processing software. According to the literature,^[^ ^49^ ^]^ the wound healing was calculated using the following Equation (4):

(4) $$\mathrm{Wound}\mathrm{healing}\mathrm{percentage}(\%)=\frac{S_{0}-S_{t}}{S_{0}}\times 100\%$$

where *S_0_* is the wound area on day 0, and *S_t_* is the wound area after a given time of treatment.

The scarless area percentage was calculated using the following Equation (5):

(5) $$\mathrm{Scarless}\mathrm{area}\mathrm{percentage}\left(\%\right)=\frac{S_{0}-S_{t}}{S_{0}}\times 100\%$$

where *S_0_* represents the wound area on day 0, *S_t_* represents the wound area on day 21.

### Cell Migration

The effect of E‐PCL degradation product on cell migration was studied in HaCaT cells. Briefly, cells were seeded on 12 well tissue culture‐treated plates. After 24 h of cell attachment, cells were serum‐starved overnight. A micro‐injury was made using a 200 µL tip and washed with PBS. Cells were treated with the degradation product of E‐PCL1 after 72 h at a concentration of 100 µg mL^−1^, and the wound closure was observed and imaged at regular intervals. The migration was studied on hours 0, 24, and 48 in HaCaT cells, respectively. The wound area was quantified using ImageJ software which was used to calculate the percentage of wound closure and migration rate.

According to the study,^[^ ^37^ ^]^ the DPPH Radical Scavenging Assay Kit (Shanghai Yuanye Bio‐Technology Co., Ltd.) was employed to evaluate the DPPH scavenging rates of 6‐HA and E‐PCL at 100 µg mL^−1^. To investigate the effect of E‐PCL1 (100 µg mL^−1^) on cellular SOD activity in HaCaT cells, oxidative stress models were established using H_2_O_2_ induction. HaCaT cells were seeded and allowed to adhere. In the Repair Group, cells were treated with 1 mm H_2_O_2_ for 4 h, followed by fresh medium replacement and 15‐h incubation. In the Protection Group, cells were first incubated in complete medium for 15 h before a 4‐h exposure to 1 mm H_2_O_2_. For treatments, the Repair Group received E‐PCL1 for 15 h after H_2_O_2_ exposure, while the Protection Group was pretreated with E‐PCL1 before H_2_O_2_ exposure. After treatment, cells were collected, lysed, and total protein was extracted for SOD activity measurement using a Total SOD Activity Assay Kit (NBT method, Beyotime Biotechnology).

### Statistical Analysis

Quantitative data were shown as mean ± standard deviation (SD). The significance between groups was assessed by unpaired Student's *t*‐test or one‐way analysis of variance with SPSS (IBM Corp.) The differences were considered significant at *p* < 0.05 (*p < 0.05; **p < 0.01; ***p <0.001). The graphs were carried out using OriginPro 2020 (Learning Edition). Histological examination was performed by capturing at least three fields per section and images were analyzed using Image J software.

## Conflict of Interest

The authors declare no conflict of interest.

## Supporting information



Supporting Information

## Data Availability

The data that support the findings of this study are available on request from the corresponding author. The data are not publicly available due to privacy or ethical restrictions.

## References

[1] a) C. Li , C. Guo , V. Fitzpatrick , A. Ibrahim , M. J. Zwierstra , P. Hanna , A. Lechtig , A. Nazarian , S. J. Lin , D. L. Kaplan , Nat. Rev. Mater. 2020, 5, 61.

[2] a) R. Dong , Y. Li , M. Chen , P. Xiao , Y. Wu , K. Zhou , Z. Zhao , B. Z. Tang , Small Methods 2022, 6, 2101247. 10.1002/smtd.202101247 35218160

[3] a) I. N. Vikhareva , E. A. Buylova , G. U. Yarmuhametova , G. K. Aminova , A. K. Mazitova , J. Chem‐NY 2021, 5099705.

[4] a) M. Ganesh , R. N. Dave , W. L'Amoreaux , R. A. Gross , Macromolecules 2009, 42, 6836.

[5] a) Y. Feng , Y. Xu , S. Liu , D. Wu , Z. Su , G. Chen , J. Liu , G. Li , Coordin. Chem. Rev. 2022, 459, 214414.

[6] a) J. Park , S. Lee , M. Lee , H.‐S. Kim , J. Y. Lee , Small 2023, 19, 2300250. 10.1002/smll.202300250 36828790

[7] a) S. Anju , N. Prajitha , V. S. Sukanya , P. V. Mohanan , Mater. Today Chem. 2020, 16, 100236.

[8] a) Z. Xu , J. Shen , L. Lin , J. Chen , L. Wang , X. Deng , X. Wu , Z. Lin , Y. Zhang , R. Yu , Z. Xu , J. Zhang , Y. Zhang , C. Wang , Environ. Int. 2023, 181, 108296. 37924603 10.1016/j.envint.2023.108296

[9] a) Z. Yuan , Y. Zhao , M. Shafiq , J. Song , J. Hou , Y. Liang , X. Yu , Y. Chen , F. Yu , M. El‐Newehy , H. El‐Hamshary , Y. Morsi , S. Jiang , H. Zheng , X. Mo , Adv. Fiber Mater 2023, 5, 1963.

[10] G. Guo , J. Hu , F. Wang , D. Fu , R. Luo , F. Zhang , C. Hu , J. Chen , X. Pan , L. Yang , Y. Wang , X. Zhang , Biomaterials 2022, 291, 121909. 36401954 10.1016/j.biomaterials.2022.121909

[11] L. Ting , L. Yongchao , L. Chan , L. Yeda , L. Dongxu , Y. Zhiming , J. Wound Care 2023, 32, 220. 37029964 10.12968/jowc.2023.32.4.220

[12] a) M. Fu , Y. Zhao , Y. Wang , Y. Li , M. Wu , Q. Liu , Z. Hou , Z. Lu , K. Wu , J. Guo , Small 2023, 19, 2205489. 10.1002/smll.202205489 36319477

[13] R. Dong , B. Guo , Nano Today 2021, 41, 101290.

[14] T. Cui , J. Yu , Q. Li , C. F. Wang , S. Chen , W. Li , G. Wang , Adv. Mater. 2020, 32, 2000982. 10.1002/adma.202000982 32627895

[15] Y. Yang , M. Li , G. Pan , J. Chen , B. Guo , Adv. Funct. Mater. 2023, 33, 2214089.

[16] a) F. M. F. Monteiro , G. M. M. Silva , J. B. R. Silva , C. S. Porto , L. B. Carvalho , J. L. L. Filho , A. M. A. Carneiro‐Leão , M. D. G. Carneiro‐DA‐Cunha , A. L. F. Porto , Process Biochem. 2007, 42, 884.

[17] a) Q. Huang , S. Kimura , T. Iwata , Polym. Degrad. Stabil. 2021, 190, 109647.

[18] M. Guicherd , M. Ben Khaled , M. Guéroult , J. Nomme , M. Dalibey , F. Grimaud , P. Alvarez , E. Kamionka , S. Gavalda , M. Noël , M. Vuillemin , E. Amillastre , D. Labourdette , G. Cioci , V. Tournier , V. Kitpreechavanich , P. Dubois , I. André , S. Duquesne , A. Marty , Nature 2024, 631, 884. 39020178 10.1038/s41586-024-07709-1

[19] X. Tian , H. Bai , Y. Zheng , L. Jiang , Adv. Funct. Mater. 2011, 21, 1398.

[20] T. Kaufman , E. H. Eichenlaub , M. F. Angel , M. Levin , J. W. Futrell , Burns 1985, 12, 84. 10.1016/0305-4179(85)90032-4 4092157

[21] M. Bartnikowski , T. R. Dargaville , S. Ivanovski , D. W. Hutmacher , Prog. Polym. Sci. 2019, 96, 1.

[22] M. S Karizmeh , S. A. Poursamar , A. Kefayat , Z. Farahbakhsh , M. Rafienia , Biomater. Adv. 2022, 135, 112667. 10.1016/j.msec.2022.112667 35577687

[23] L. Yin , Q. Tang , Q. Ke , X. Zhang , J. Su , H. Zhong , L. Fang , ACS Appl. Mater. Interfaces 2023, 15, 48903. 37877332 10.1021/acsami.3c09584

[24] A. D. Vethaak , J. Legler , Science 2021, 371, 672. 33574197 10.1126/science.abe5041

[25] H. Liu , R. Chen , P. Wang , J. Fu , Z. Tang , J. Xie , Y. Ning , J. Gao , Q. Zhong , X. Pan , D. Wang , M. Lei , X. Li , Y. Zhang , J. Wang , H. Cheng , Carbohyd. Polym. 2023, 316, 121050. 10.1016/j.carbpol.2023.121050 37321740

[26] L. Huang , L. Gao , C. Chen , Trends Endocrin. Met. 2021, 32, 351. 10.1016/j.tem.2021.03.002 33832826

[27] R. Wen , C. Zhou , J. Tian , J. Lu , Talanta 2023, 252, 123830. 36030738 10.1016/j.talanta.2022.123830

[28] M. Z. Kamal , P. Yedavalli , M. V. Deshmukh , N. M. Rao , Protein Sci. 2013, 22, 904. 23625694 10.1002/pro.2271 PMC3719085

[29] L. Miranda‐Calderon , C. Yus , C. Remirez de Ganuza , M. Paesa , G. Landa , E. Tapia , E. Pérez , M. Perez , V. Sebastian , S. Irusta , G. Mendoza , M. Arruebo , Chem. Eng. J. 2023, 476, 146679.

[30] A. Orchel , K. Jelonek , J. Kasperczyk , Z. Dzierzewicz , Acta. Pol. Pharm. 2010, 67, 710. 21229893

[31] Y. Wang , Y. Zhang , Z. Lin , T. Huang , W. Li , W. Gong , Y. Guo , J. Su , J. Wang , Q. Tu , Compos. Part B: Eng 2021, 222, 109047.

[32] W. Liang , M. Jiang , J. Zhang , X. Dou , Y. Zhou , Y. Jiang , L. Zhao , M. Lang , J. Mater. Sci. Technol. 2021, 89, 225.

[33] P. Patil , K. A. Russo , J. T. McCune , A. C. Pollins , M. A. Cottam , B. R. Dollinger , C. R. DeJulius , M. K. Gupta , R. D'Arcy , J. M. Colazo , F. Yu , M. G. Bezold , J. R. Martin , N. L. Cardwell , J. M. Davidson , C. M. Thompson , A. Barbul , A. H. Hasty , S. A. Guelcher , C. L. Duvall , Sci. Transl. Med. 2022, 14, abm6586. 10.1126/scitranslmed.abm6586 PMC10165619 35442705

[34] A. Gautam , V. Kumar , L. Azmi , C. V. Rao , M. M. Khan , B. Mukhtar , M. Kamal , M. Arif , S. Mehdi , S. M. Alsanad , O. A. Al‐Khamees , T. Jawaid , A. Alam , Separations 2023, 10, 166.

[35] T. H. Chen , C. C. Chang , J. Y. Houng , T. H. Chang , Y. L. Chen , C. C. Hsu , L. S. Chang , Pharmaceuticals 2022, 15, 826.35890125

[36] Y. Zhao , X. Wu , C. Wu , R. Meng , Y. Gu , X. Xiao , Food Biotechnol. 2022, 36, 266.

[37] M. N. Sarian , Q. U. Ahmed , S. Z. Mat So'ad , A. M. Alhassan , S. Murugesu , V. Perumal , S. N. A. Syed Mohamad , A. Khatib , J. Latip , Biomed Res. Int. 2017, 8386065. 29318154 10.1155/2017/8386065 PMC5727842

[38] S. Agarwal , M. Sorkin , B. Levi , Clin. Plast. Surg. 2017, 44, 749. 28888300 10.1016/j.cps.2017.05.006 PMC5658026

[39] K. Chen , H. Pan , D. Ji , Y. Li , H. Duan , W. Pan , Mat. Sci. Eng. 2021, 127, 112245. 10.1016/j.msec.2021.112245 34225884

[40] Z. Zhou , T. Deng , M. Tao , L. Lin , L. Sun , X. Song , D. Gao , J. Li , Z. Wang , X. Wang , J. Li , Z. Jiang , L. Luo , L. Yang , M. Wu , Biomaterials 2023, 299, 122141. 37167893 10.1016/j.biomaterials.2023.122141

[41] M. A. Karsdal , S. H. Nielsen , D. J. Leeming , L. L. Langholm , M. J. Nielsen , T. Manon‐Jensen , A. Siebuhr , N. S. Gudmann , S. Rønnow , J. M. Sand , S. J. Daniels , J. H. Mortensen , D. Schuppan , Adv. Drug Delivery Rev. 2017, 121, 43. 10.1016/j.addr.2017.07.014 28736303

[42] Z. Chen , L. Xiao , C. Hu , Z. Shen , E. Zhou , S. Zhang , Y. Wang , Acta Biomater. 2023, 164, 240. 37075962 10.1016/j.actbio.2023.04.015

[43] B. Panganiban , B. Qiao , T. Jiang , C. DelRe , M. M. Obadia , T. D. Nguyen , A. A. A. Smith , A. Hall , I. Sit , M. G. Crosby , P. B. Dennis , E. Drockenmuller , M. Olvera de la Cruz , T. Xu , Science 2018, 359, 1239. 29590071 10.1126/science.aao0335

[44] K. Lu , K. Li , M. Zhang , Z. Fang , P. Wu , L. Feng , K. Deng , C. Yu , Y. Deng , Y. Xiao , P. Zhu , R. Guo , Chem. Eng. J. 2021, 424, 130429.

[45] J. D. Schrag , Y. Li , M. Cygler , D. Lang , T. Burgdorf , H.‐J. Hecht , R. Schmid , D. Schomburg , T. J. Rydel , J. D. Oliver , L. C. Strickland , C. M. Dunaway , S. B. Larson , J. Day , A. McPherson , Structure 1997, 5, 187. 9032074 10.1016/s0969-2126(97)00178-0

[46] H. Park , P. Bradley , P. Greisen Jr. , Y. Liu , V. K. Mulligan , D. E. Kim , D. Baker , F. DiMaio , J. Chem. Theory Comput. 2016, 12, 6201. 27766851 10.1021/acs.jctc.6b00819 PMC5515585

[47] a) J. Eberhardt , D. Santos‐Martins , A. F. Tillack , S. Forli , J. Chem. Inf. Model. 2021, 61, 3891. 34278794 10.1021/acs.jcim.1c00203 PMC10683950

[48] a) J. Kucera , M. Sojka , V. Pavlik , K. Szuszkiewicz , V. Velebny , P. Klein , J. Microbiol. Meth. 2014, 103, 18. 10.1016/j.mimet.2014.05.008 24880129

[49] G. C. Limandjaja , J. M. Belien , R. J. Scheper , F. B. Niessen , S. Gibbs , Brit. J. Dermatol. 2020, 182, 974. 31206605 10.1111/bjd.18219

