# Supplementary material for: Robust Controlled Degradation of Enzyme Loaded PCL‐Based Fibrous Scaffolds Toward Scarless Skin Tissue Regeneration
Source: Adv Sci (Weinh). 2025 Apr 3;12(25):2501053. doi: 10.1002/advs.202501053 (PMC12225112; doi:10.1002/advs.202501053)
Supplement: Supplementary file 1 — Supporting Information [file ADVS-12-2501053-s001.docx]

Supporting Information

**Robust Controlled Degradation of Enzyme Loaded PCL-based Fibrous Scaffolds towards Scarless Skin Tissue Regeneration**

*Lingling Fan^1^, Weiliang Dong^1,2^*, Jianqi Lu^1^, Yujia Peng^1^, Bin Xie^1^, Ping Wei^1^,*

*Min Jiang^1,2^*, Su Chen^2,3^**

1 Key Laboratory for Waste Plastics Biocatalytic Degradation and Recycling, College of Biotechnology and Pharmaceutical Engineering, Nanjing Tech University, Nanjing, China

2 State Key Laboratory of Materials-Oriented Chemical Engineering, Nanjing Tech University, Nanjing, China

3 College of Chemical Engineering, Nanjing Tech University, Nanjing, China

*Corresponding authors:

E-mail addresses: dwl@njtech.edu.cn (W.L. Dong); bioengine@njtech.edu.cn (M. Jiang); chensu@njtech.edu.cn (S. Chen).

**Supporting Figures**


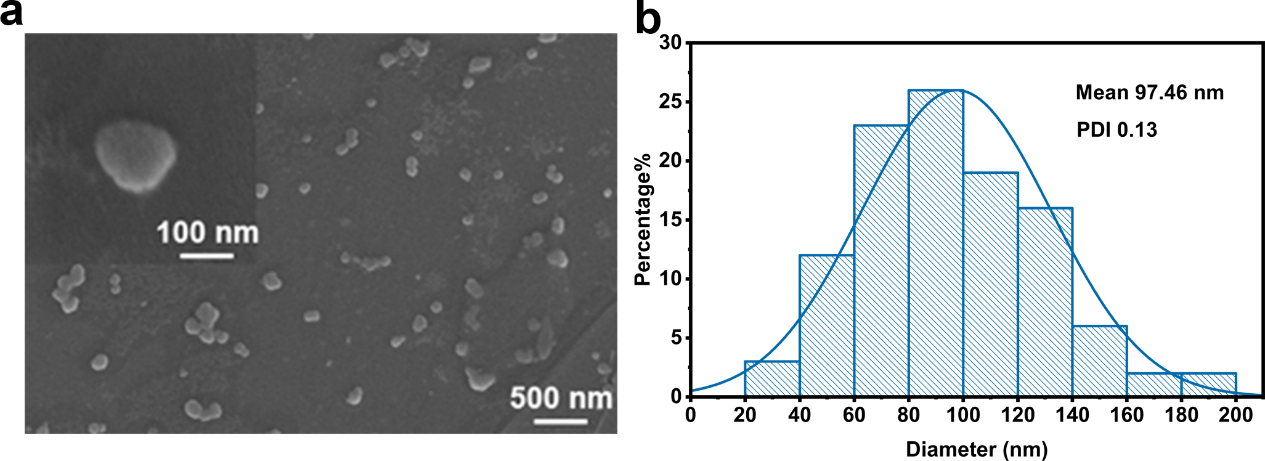


**Figure S1** a) Representative SEM images of enzyme composites in TFE. b) Corresponding particle size distribution in TFE.


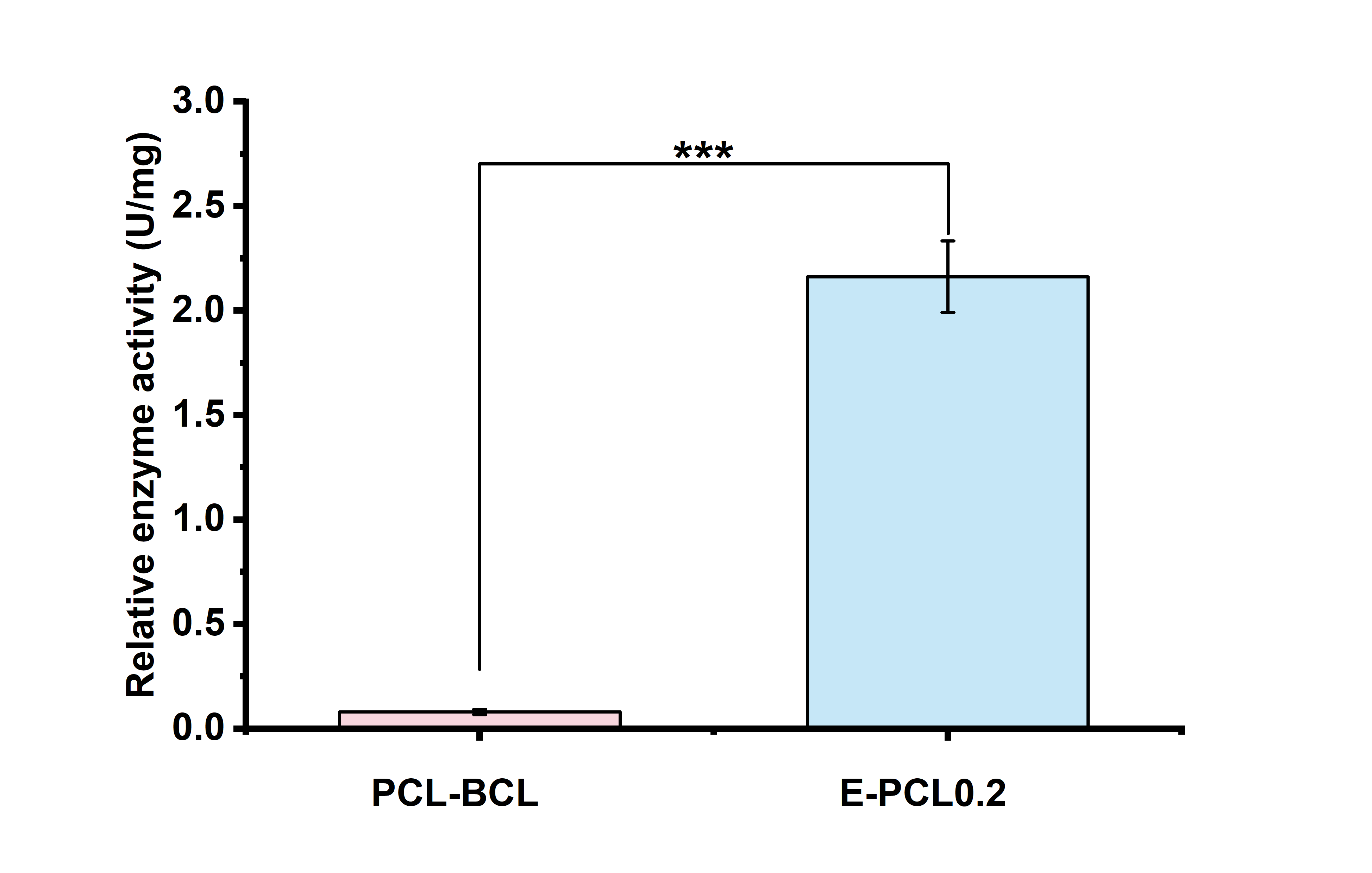


**Figure S2** Enzyme activity of PCL-BCL and E-PCL0.2 against pNP-C6.


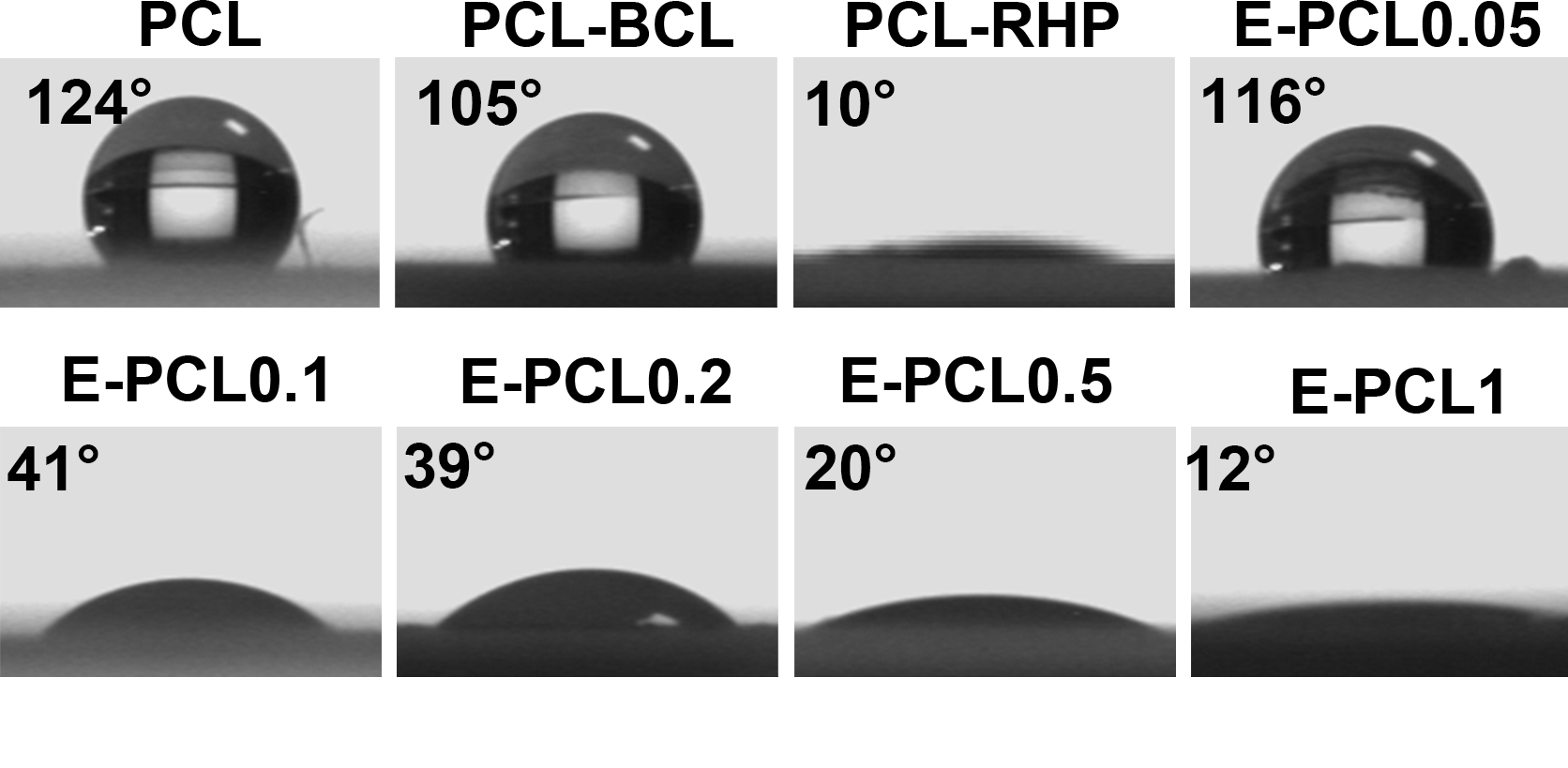


**Figure S3** Representative images of water contact angles on PCL, PCL-BCL, and E-PCLs with 0.05% (E-PCL0.05), 0.1% (E-PCL0.1), 0.2% (E-PCL0.2), 0.5% (E-PCL0.5), and 1% (E-PCL1).


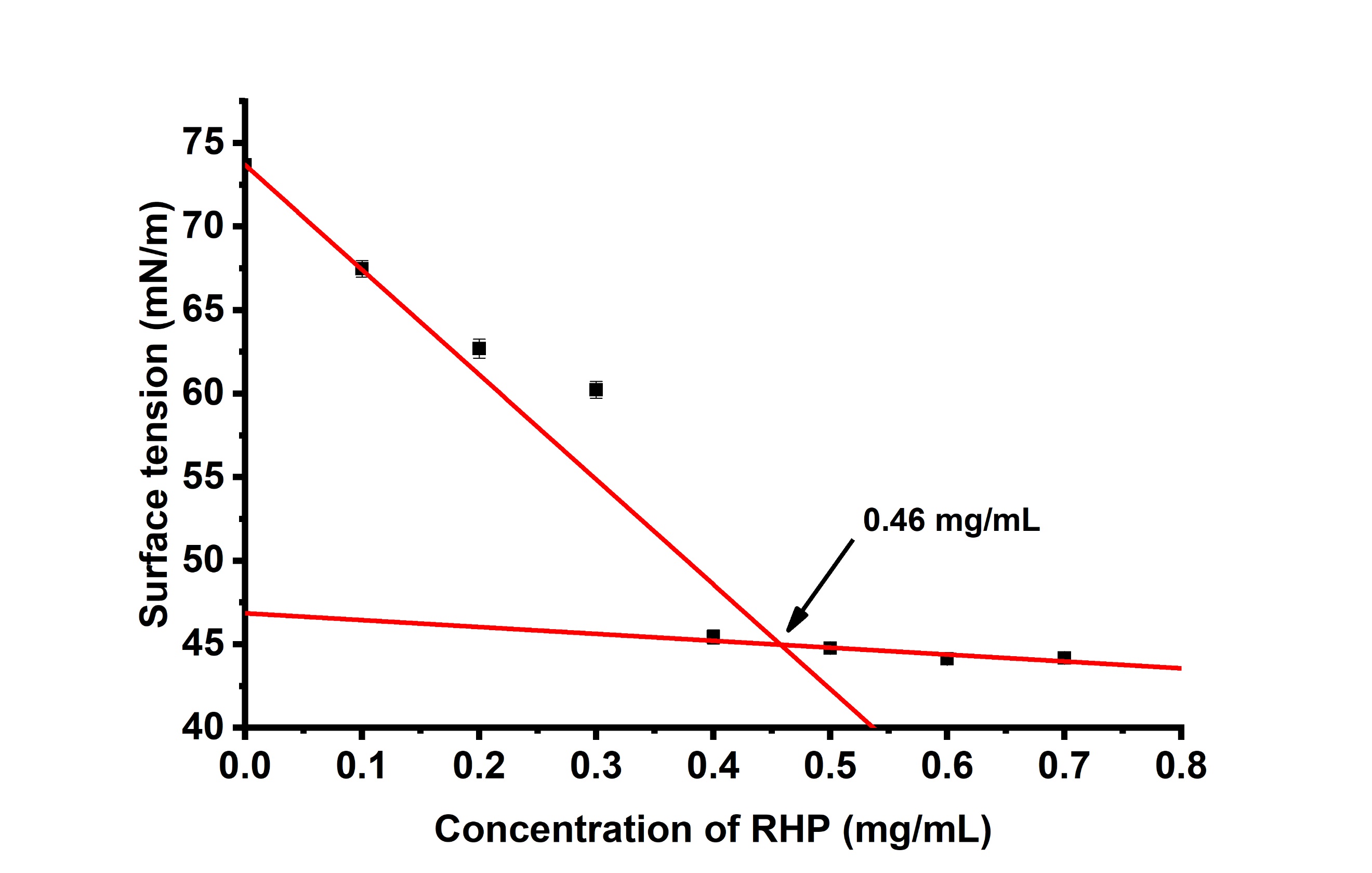


**Figure S4** Surface tension measurements with water of RHP.


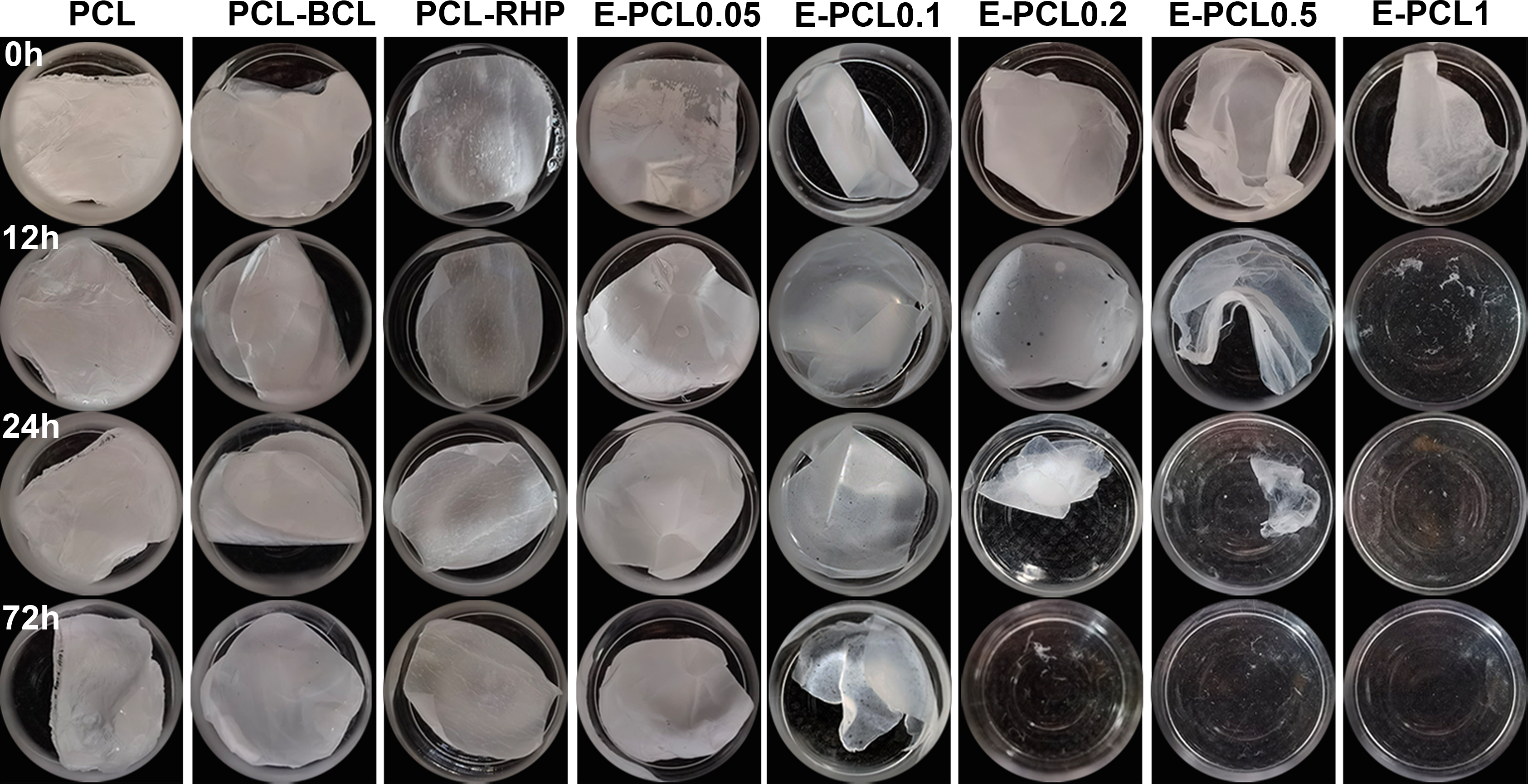


**Figure S5** Representative digital images of the degradation process of PCL, PCL-BCL, and PCL-RHP as control groups, alongside the experimental groups E-PCLs (E-PCL0.05, E-PCL0.1, E-PCL0.2, E-PCL0.5, and E-PCL1). Samples were observed in 50 mM PB (pH 7.0) on hour 0, 12, 24, and 72, respectively.


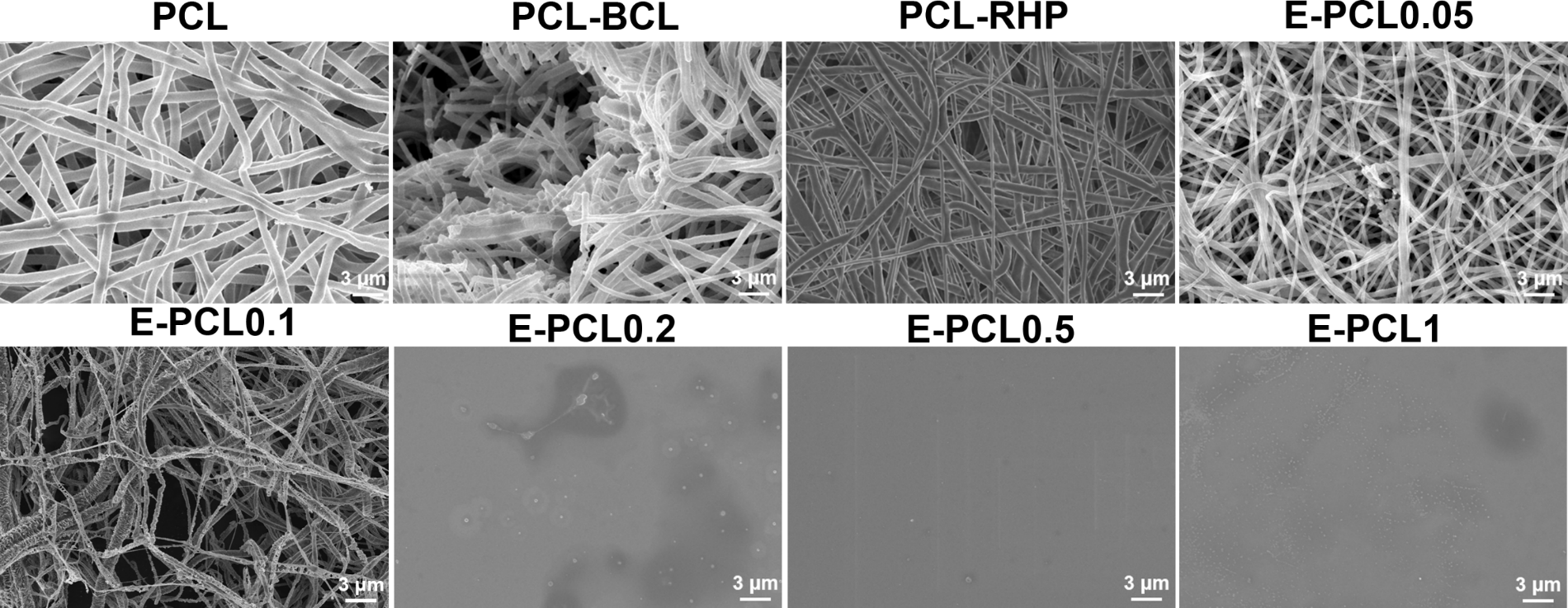


**Figure S6** Representative SEM images of the degradable scaffolds on hour 72.


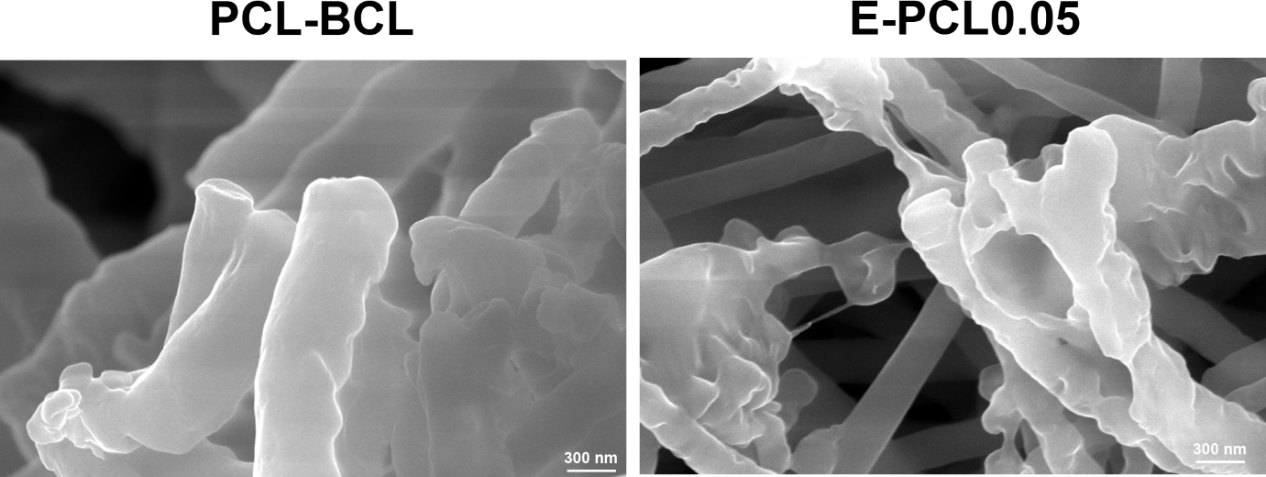


**Figure S7** Representative SEM images of the degradable scaffolds (PCL-BCL and E-PCL0.05) on hour 72.


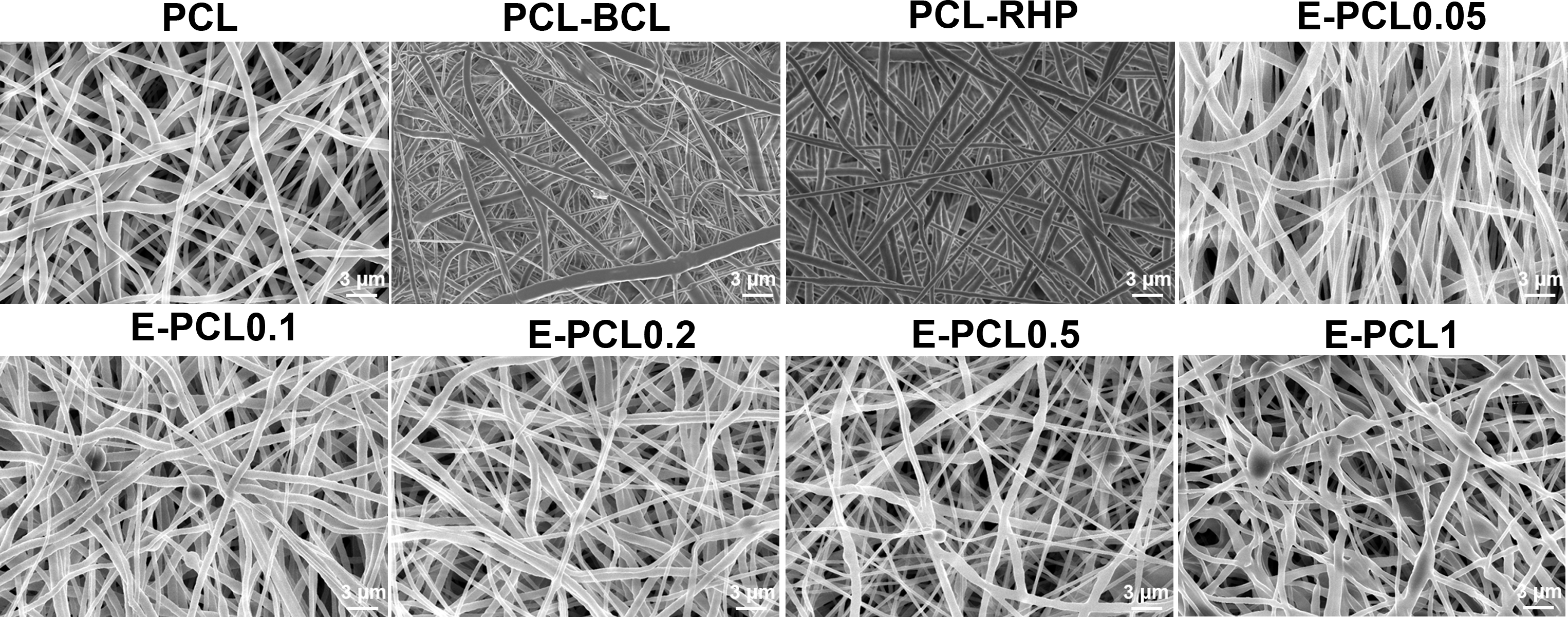


**Figure S8** Representative SEM images of the original scaffolds.


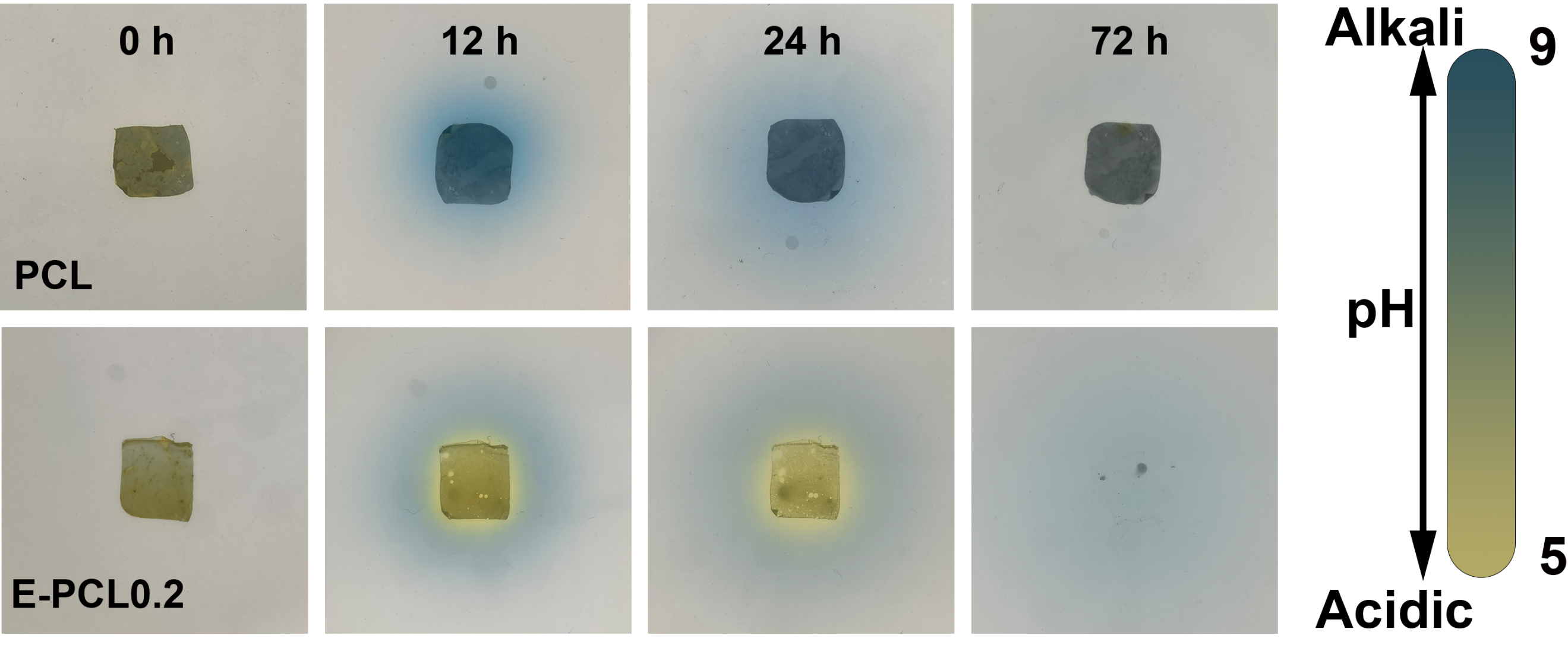


**Figure S9** Representative images of E-PCL0.2 containing 0.5wt% bromothymol blue on non-buffered plates at pH 9.


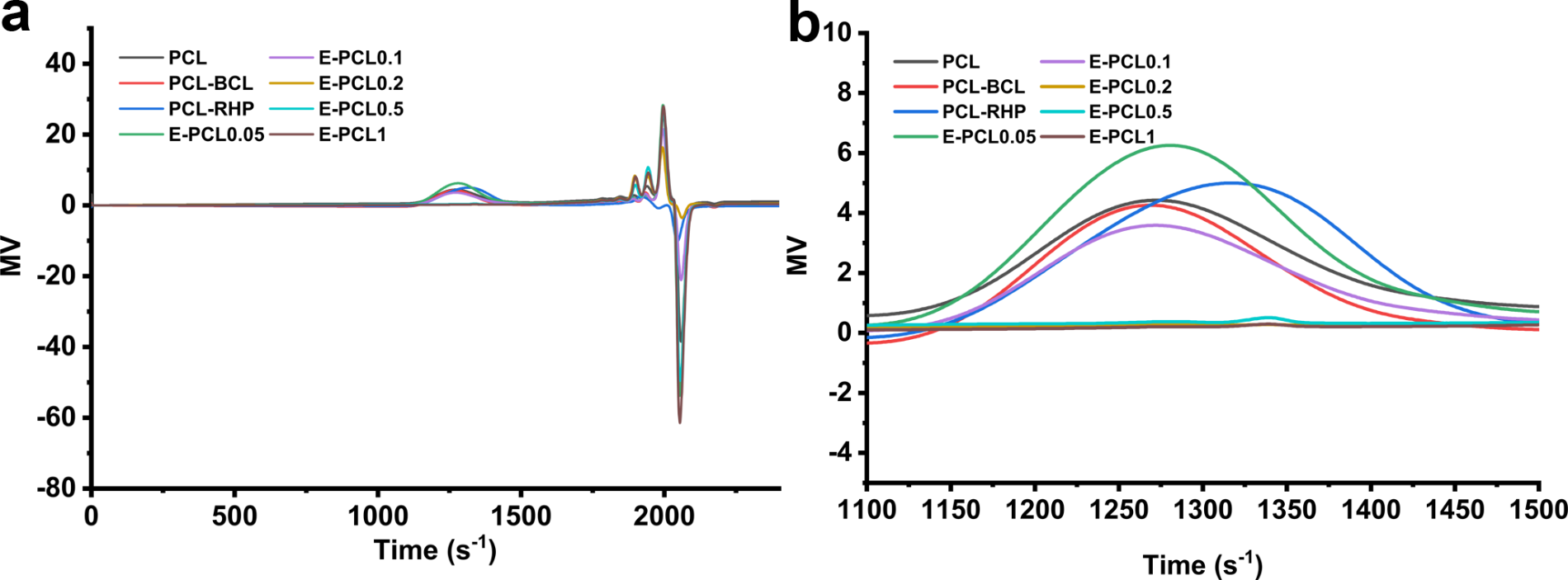


**Figure S10** a) The GPC results of scaffolds. b) The graph of local amplification from the 1100s to the 1500s.


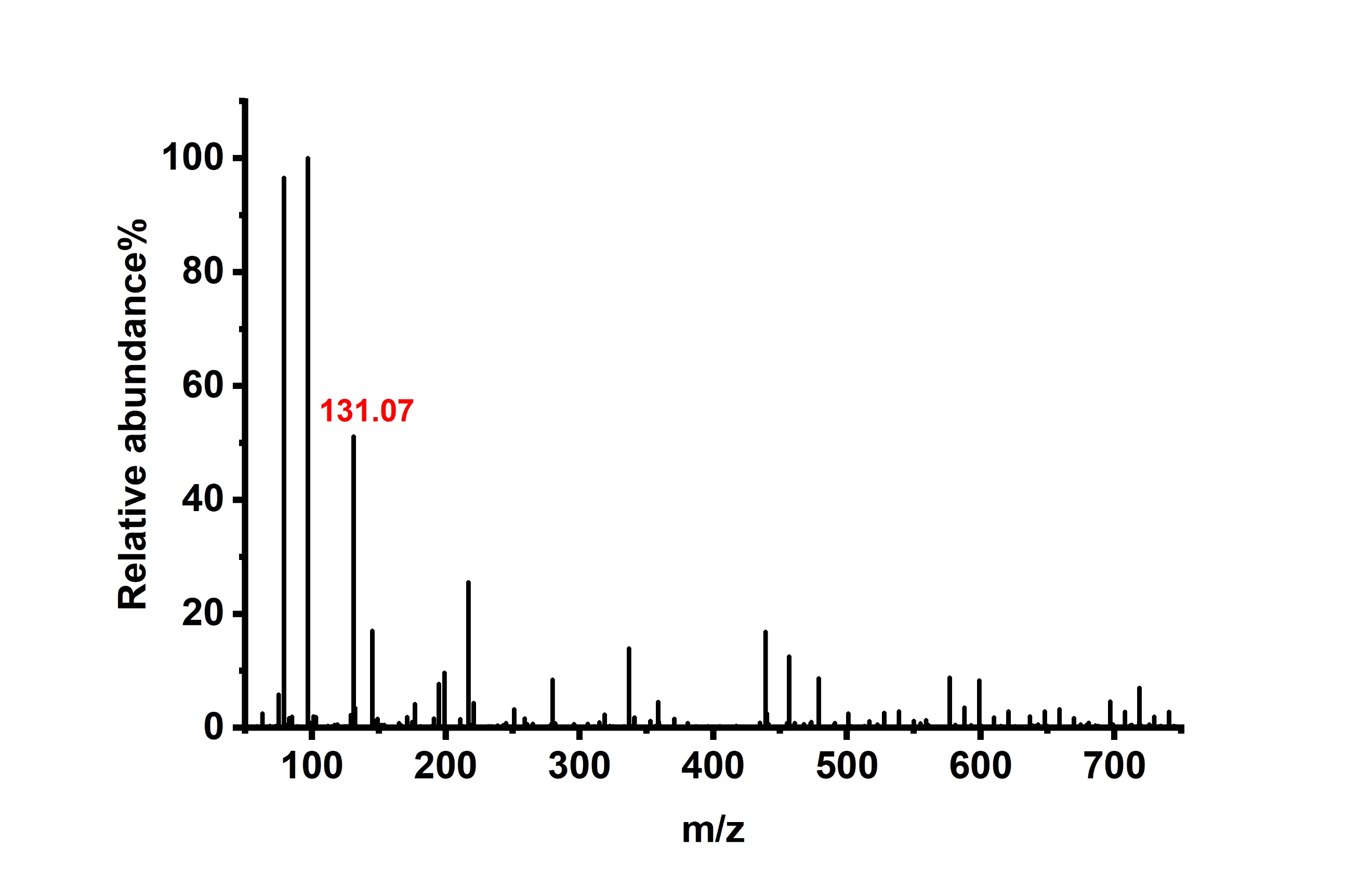


**Figure S11** The MS data of the E-PCL1 degradation solution on hour 96.


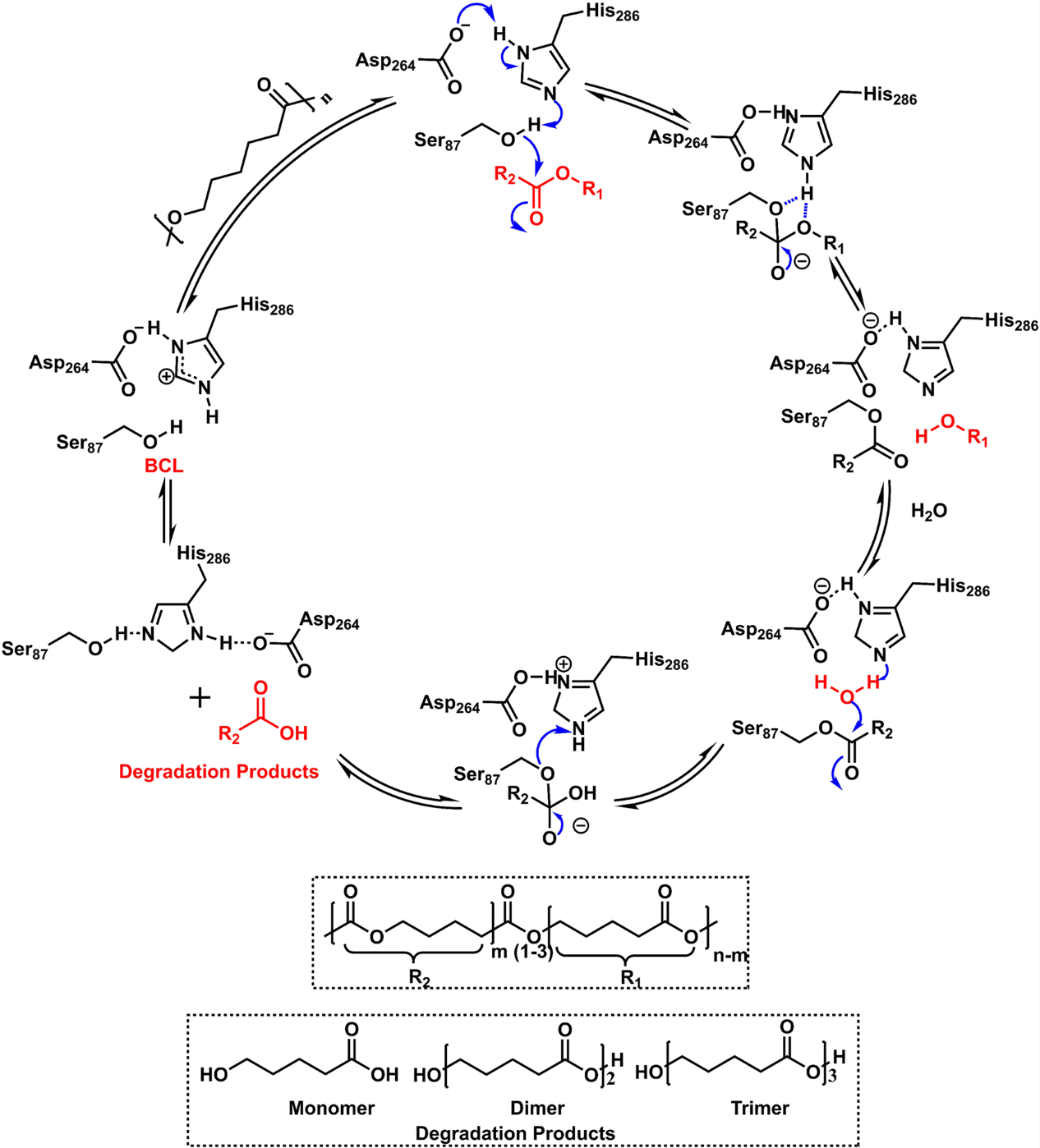


**Figure S12** Proposed mechanism of lipase-catalyzed cleavage of PCL.


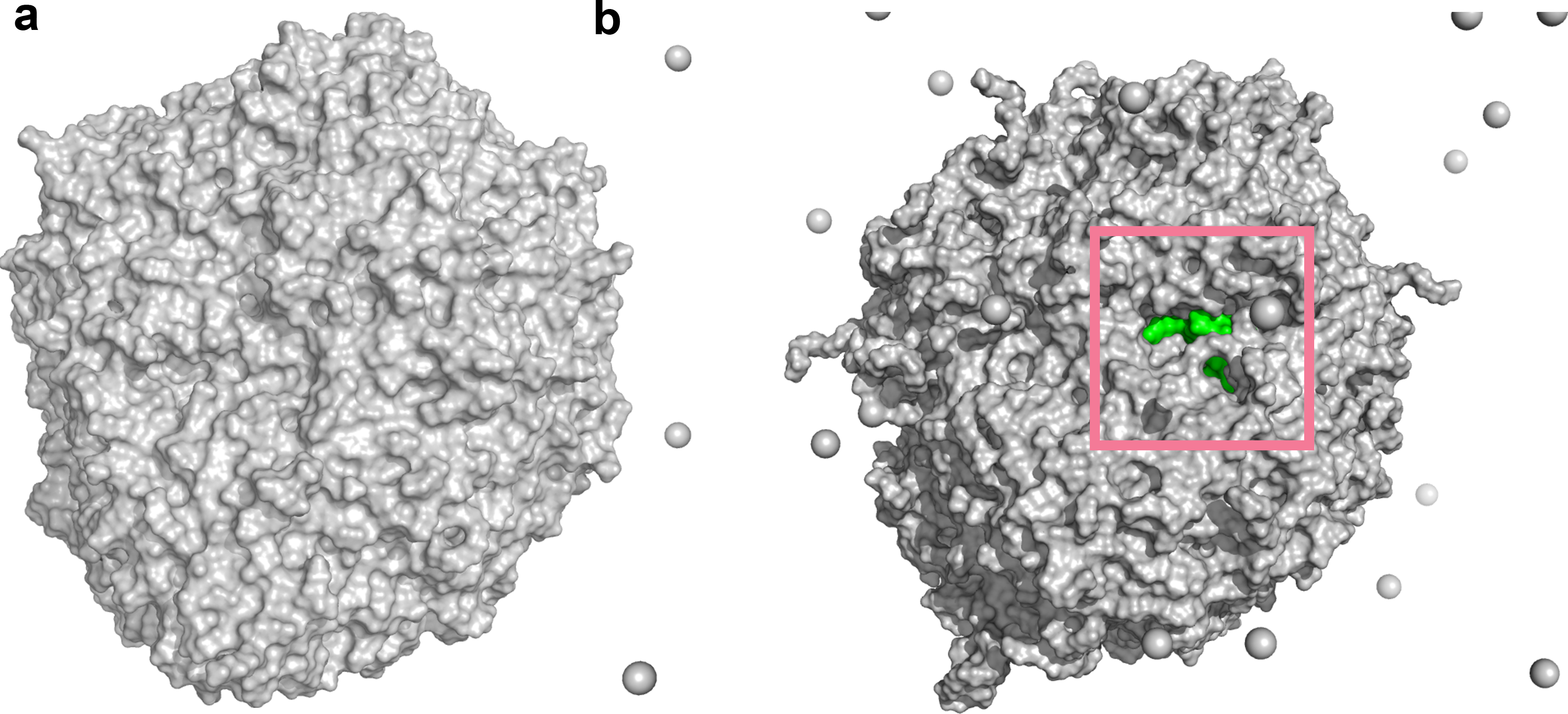


**Figure S13** a) Initial 3D structure for molecular dynamic simulation in water. b) 3D structure of an enzyme composite after 40 ns at 400K and 200 ns at 310K in water. Green represents BCL, and grey represents RHP.


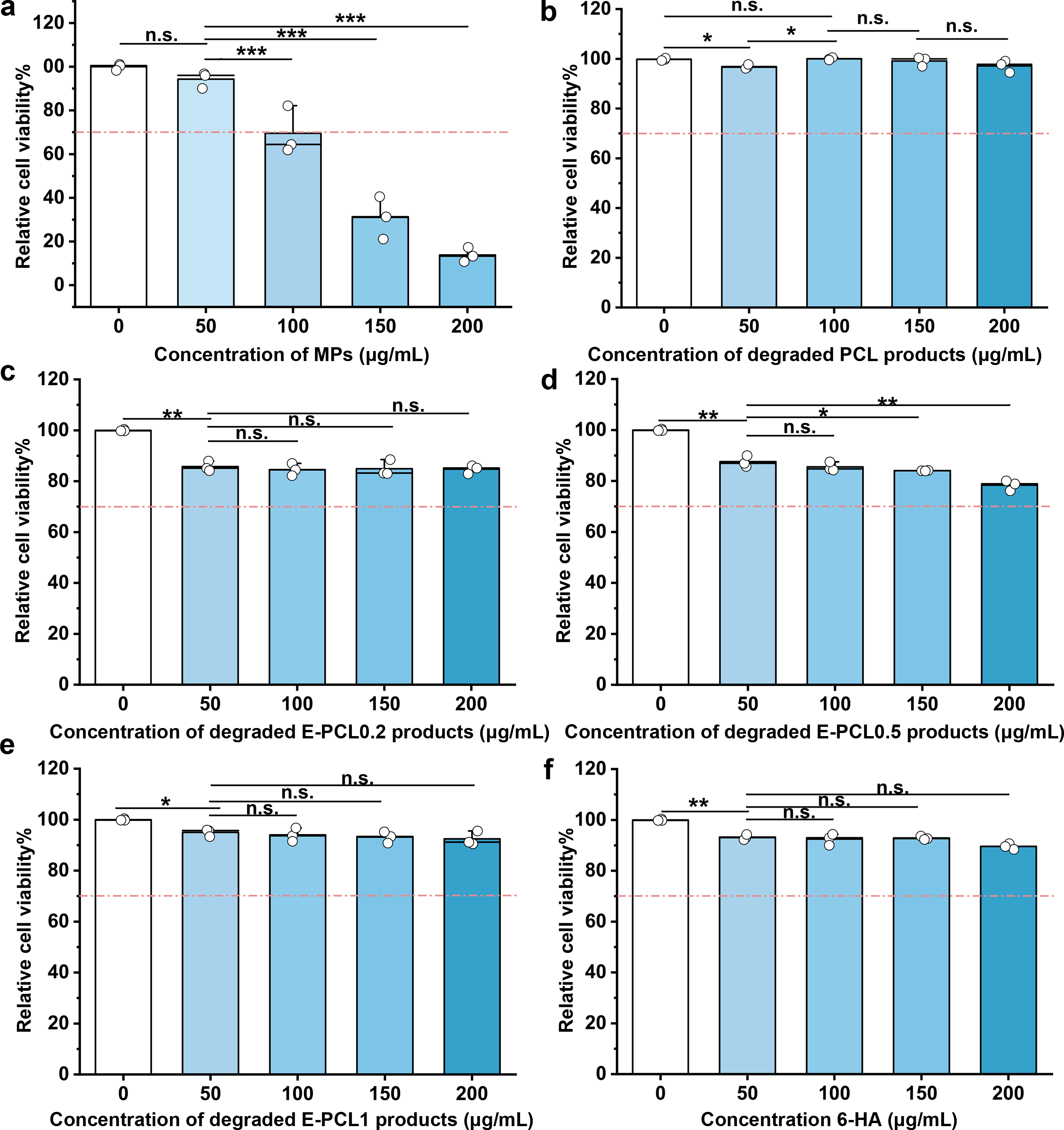


**Figure S14** The relative cell viability results of the CCK-8 assay with L929 cells of degraded samples’ products. a) MPs, b) PCL, c) E-PCL0.2, d) E-PCL0.5, and e) E-PCL1, and f) 6-HA, respectively. E-PCLs represent the degradation products on hour 72.


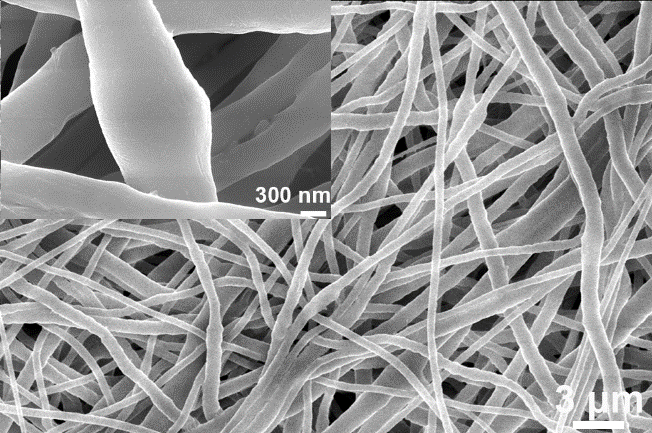


**Figure S15** Representative SEM images of E-PCL0.2 after three months of exposure to air at 37°C.


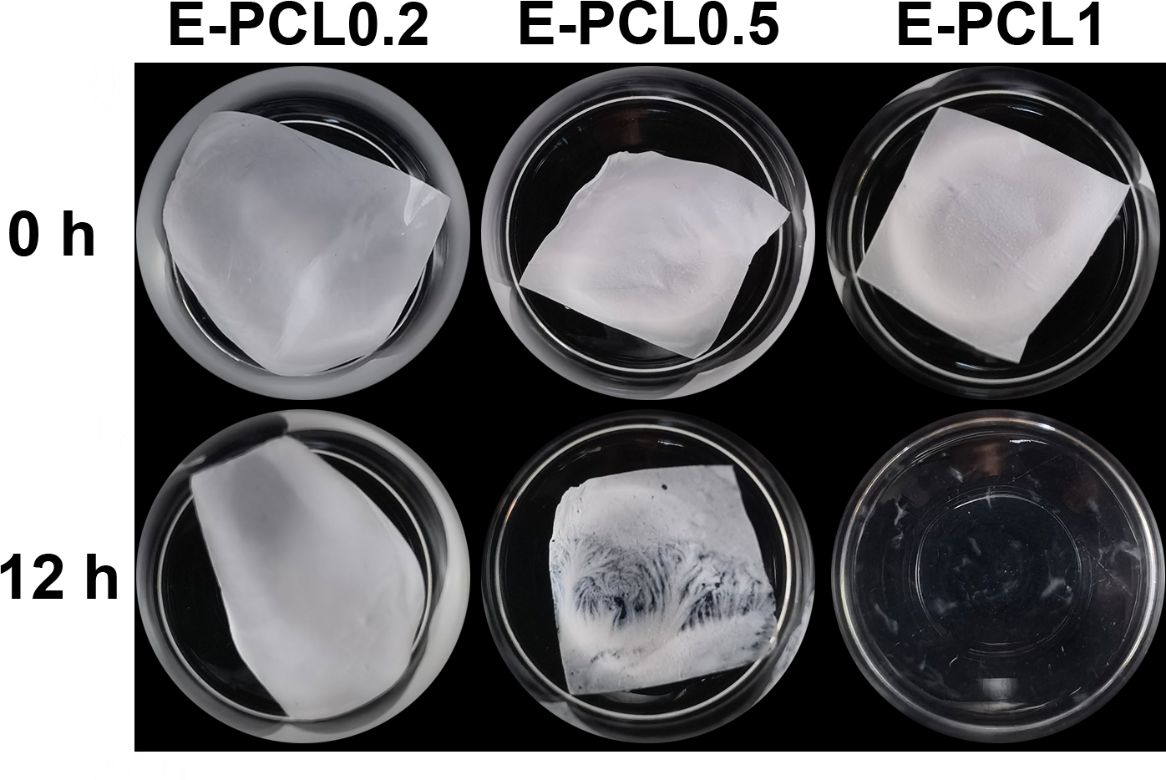


**Figure S16** Representative digital images of E-PCL0.2, E-PCL0.5, and E-PCL1 degraded in PB on hour 0 and 12, respectively. These samples were stored at room temperature for one year.


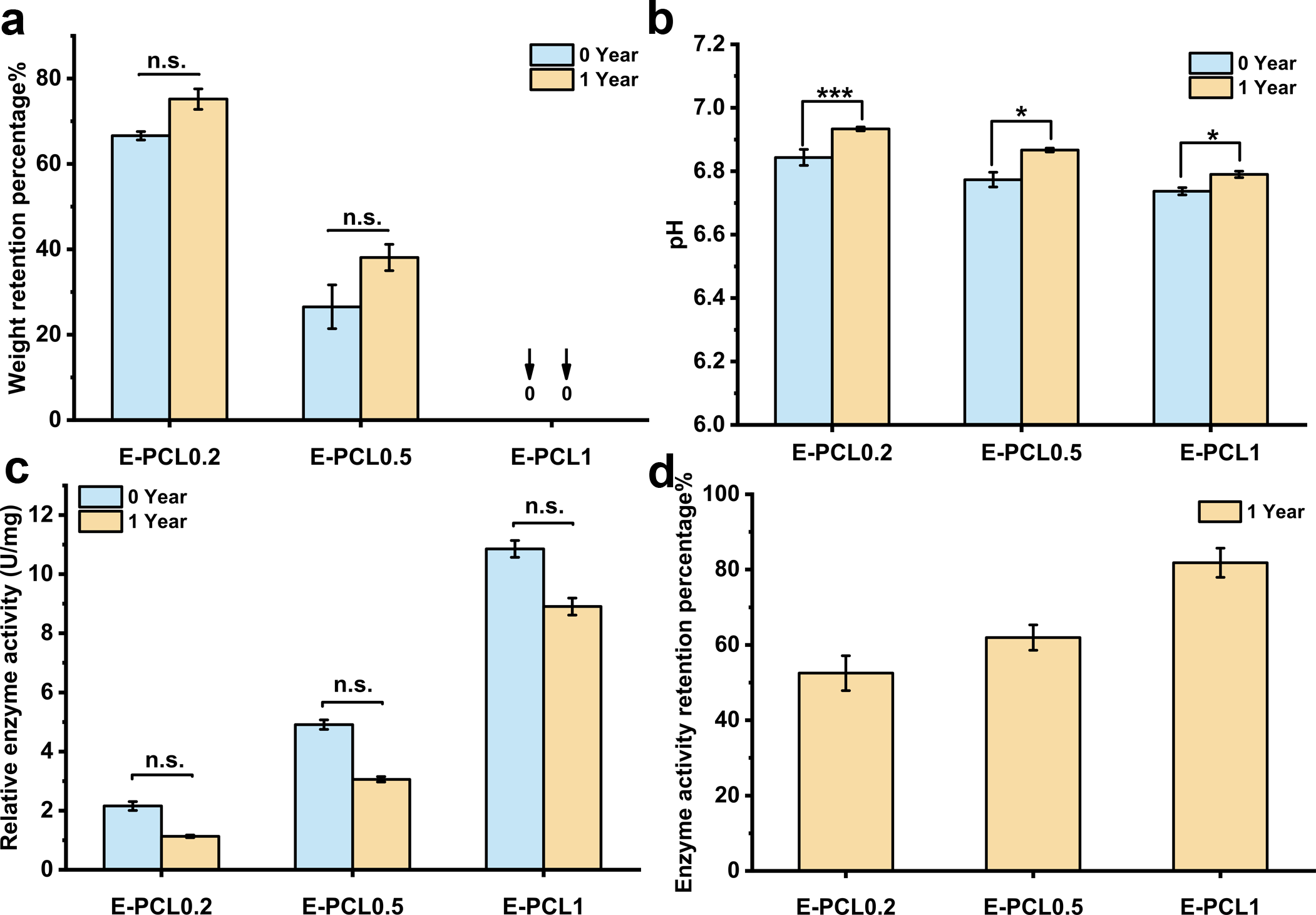


**Figure S17** Graph illustrating a) weight retention percentage, b) pH value, c) relative enzyme activity, and d) enzyme activity retention rate for E-PCL0.2, E-PCL0.5, and E-PCL1. These samples were stored at room temperature for one year before degradation in phosphate buffer.


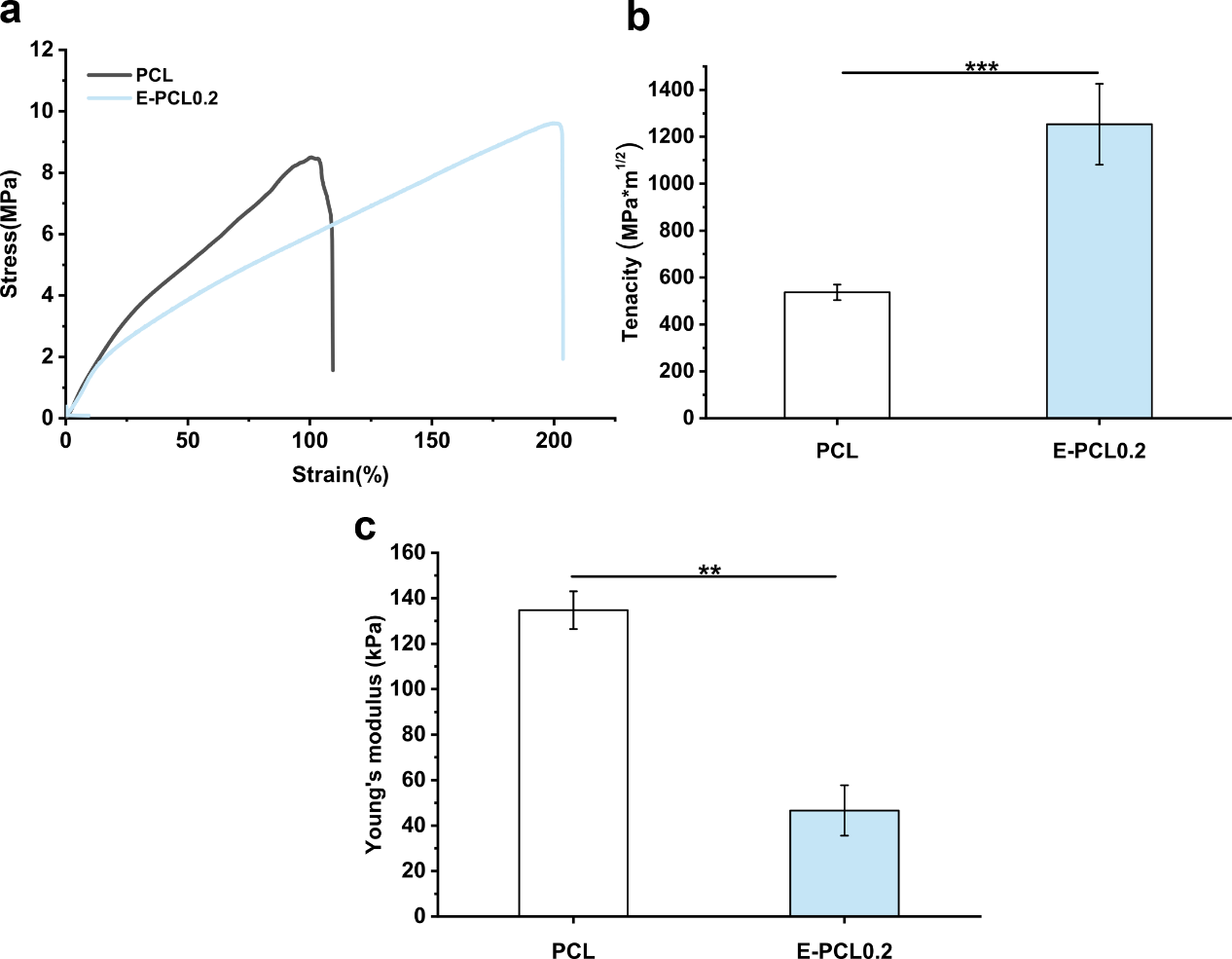


**Figure S18** Graph illustrating a) tensile stress-strain curves, b) tenacity, and c) Young’s modulus of PCL and E-PCL0.2


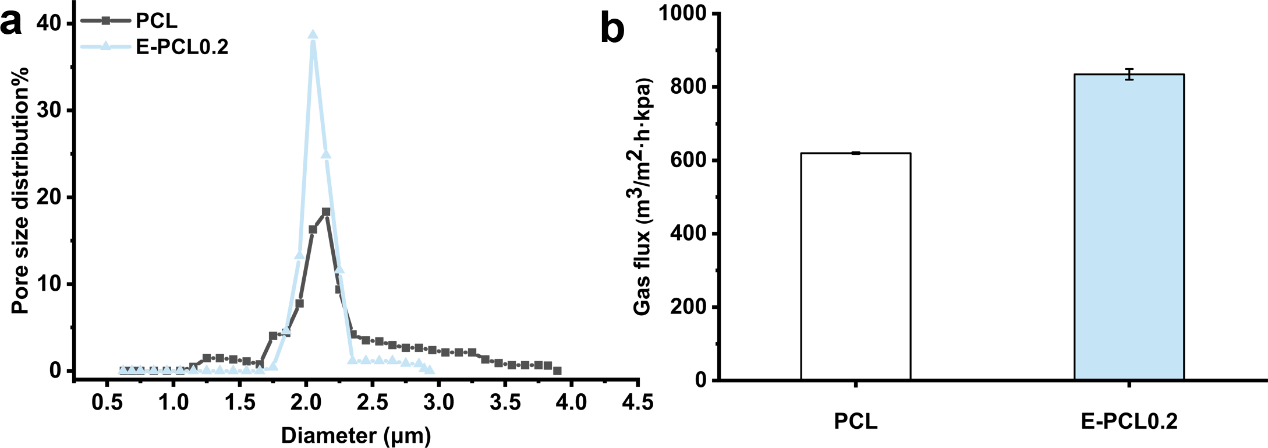


**Figure S19** Graph illustrating a) pore size diameter and b) gas flux data of PCL and E-PCL0.2.


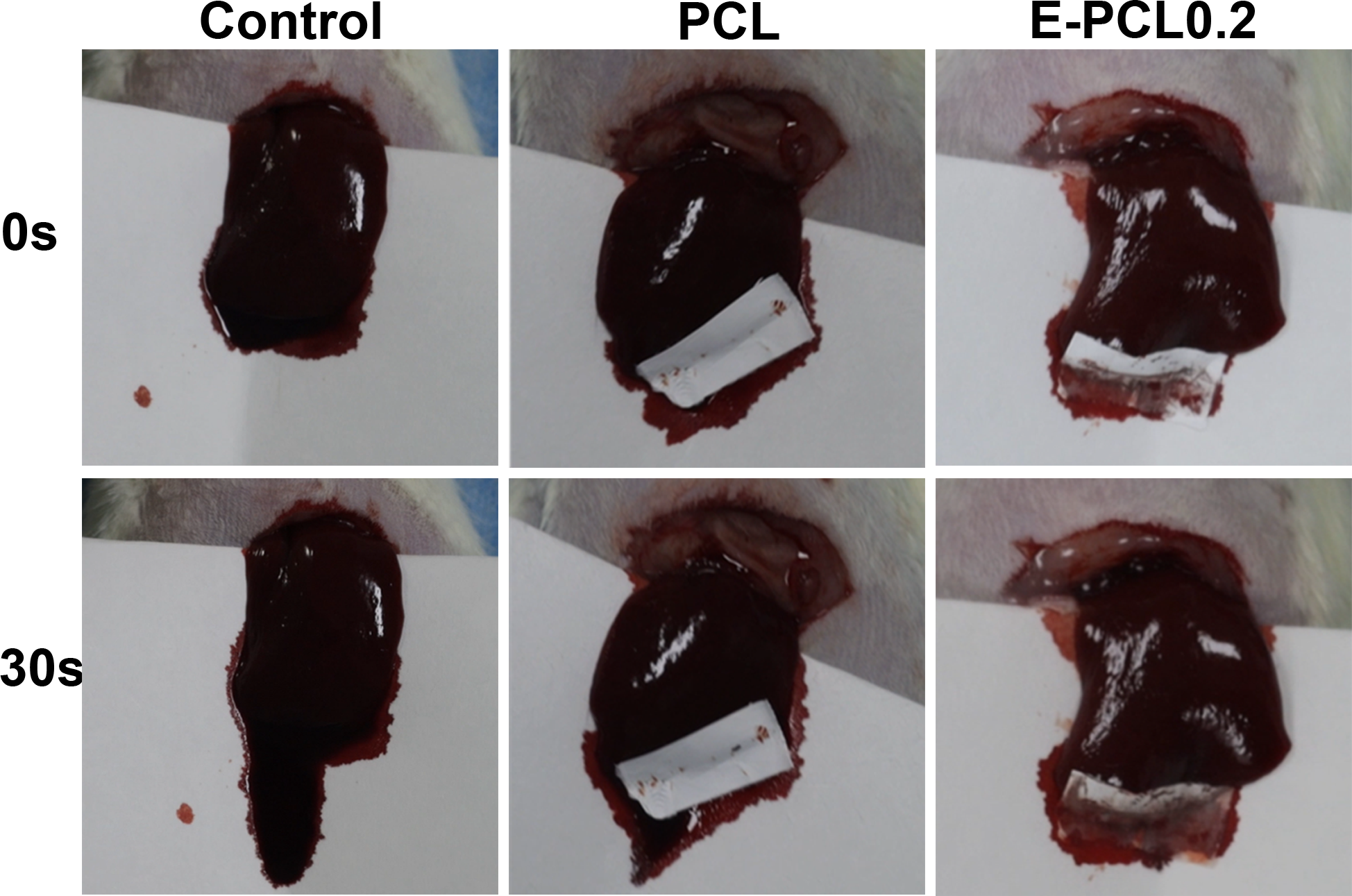


**Figure S20** Representative image of *in vivo* hemostasis of control, PCL and E-PCL0.2 on seconds 0 and 30, respectively.


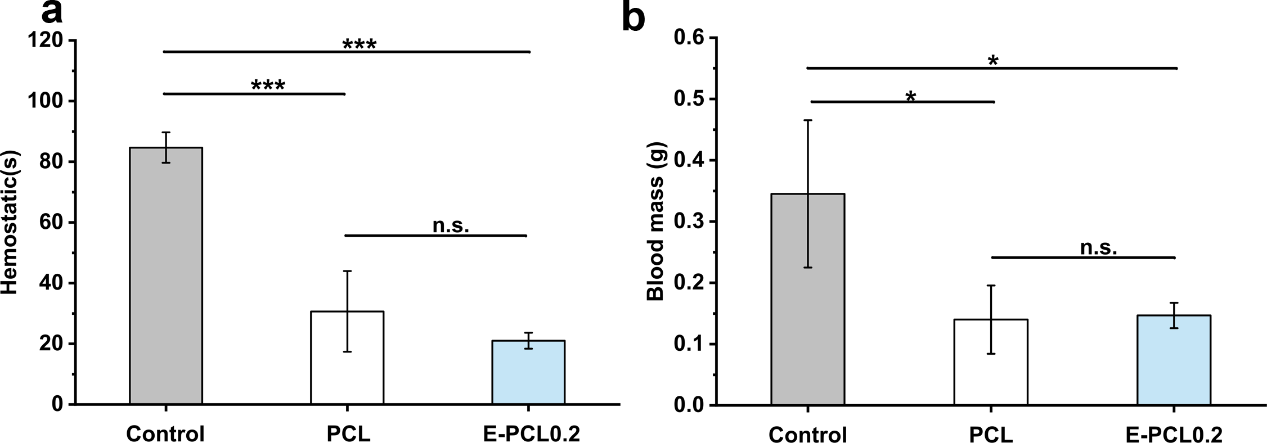


**Figure S21** Graph illustrating a) hemostatic time b) blood mass of control, PCL, and E-PCL0.2 group, respectively.


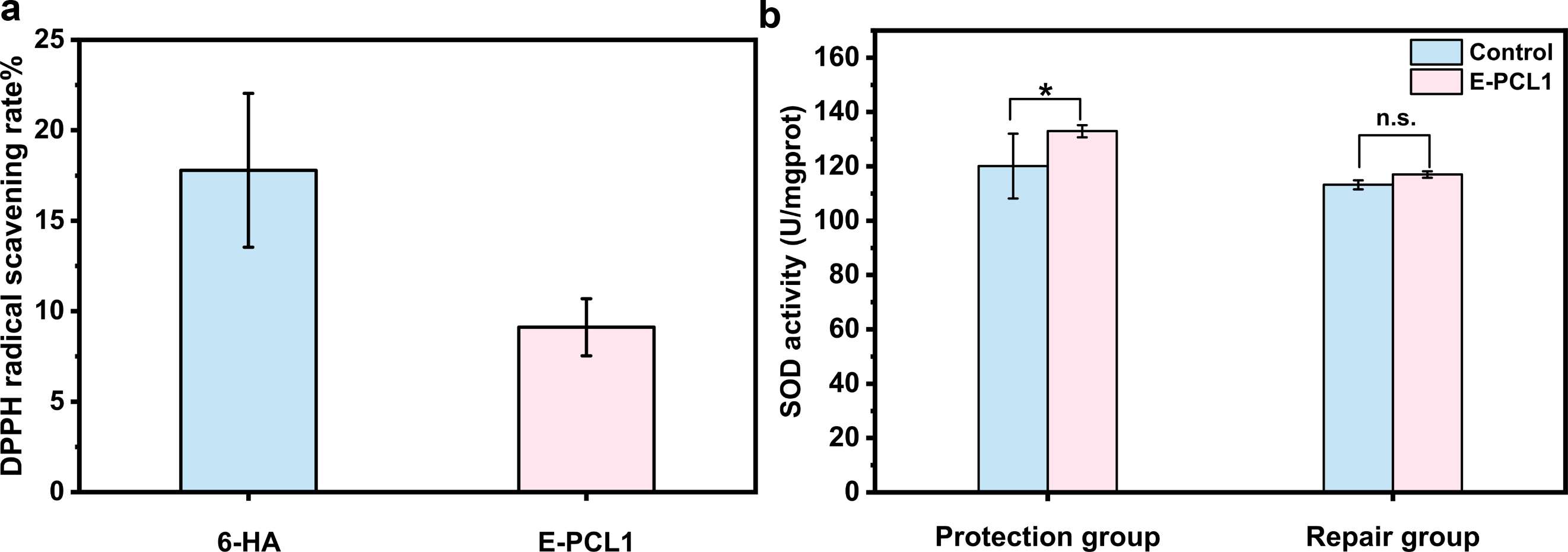


**Figure S22** a) Scavenging ability of DPPH of 6-HA and E-PC1 at 100 μg/mL. b) Impact on cellular SOD activity by E-PCL1 (100 μg/mL). The control group was the H_2_O_2_ model group


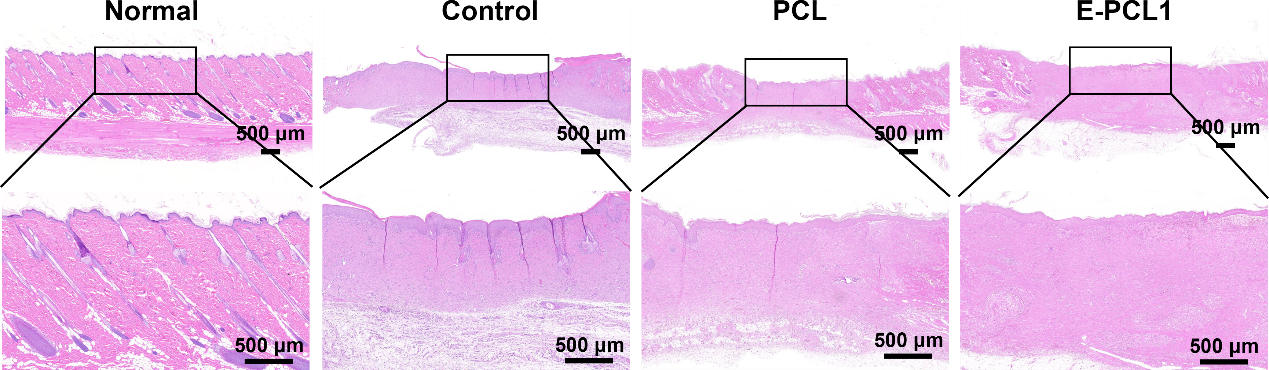


**Fig. S23** Representative images of the H&E staining on day 14.


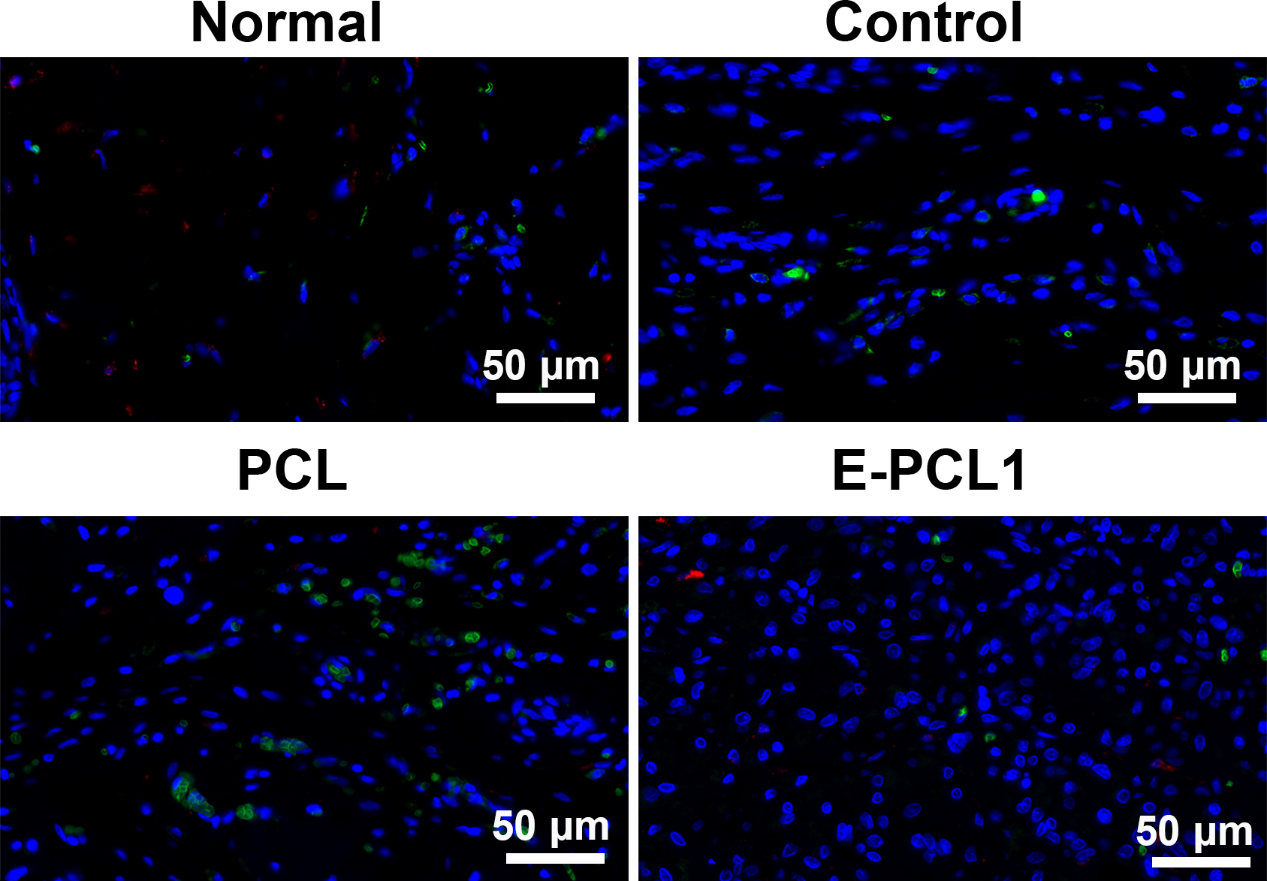


**Figure S24** Representative images of the CD86/CD206 immunohistochemical staining on day 14.


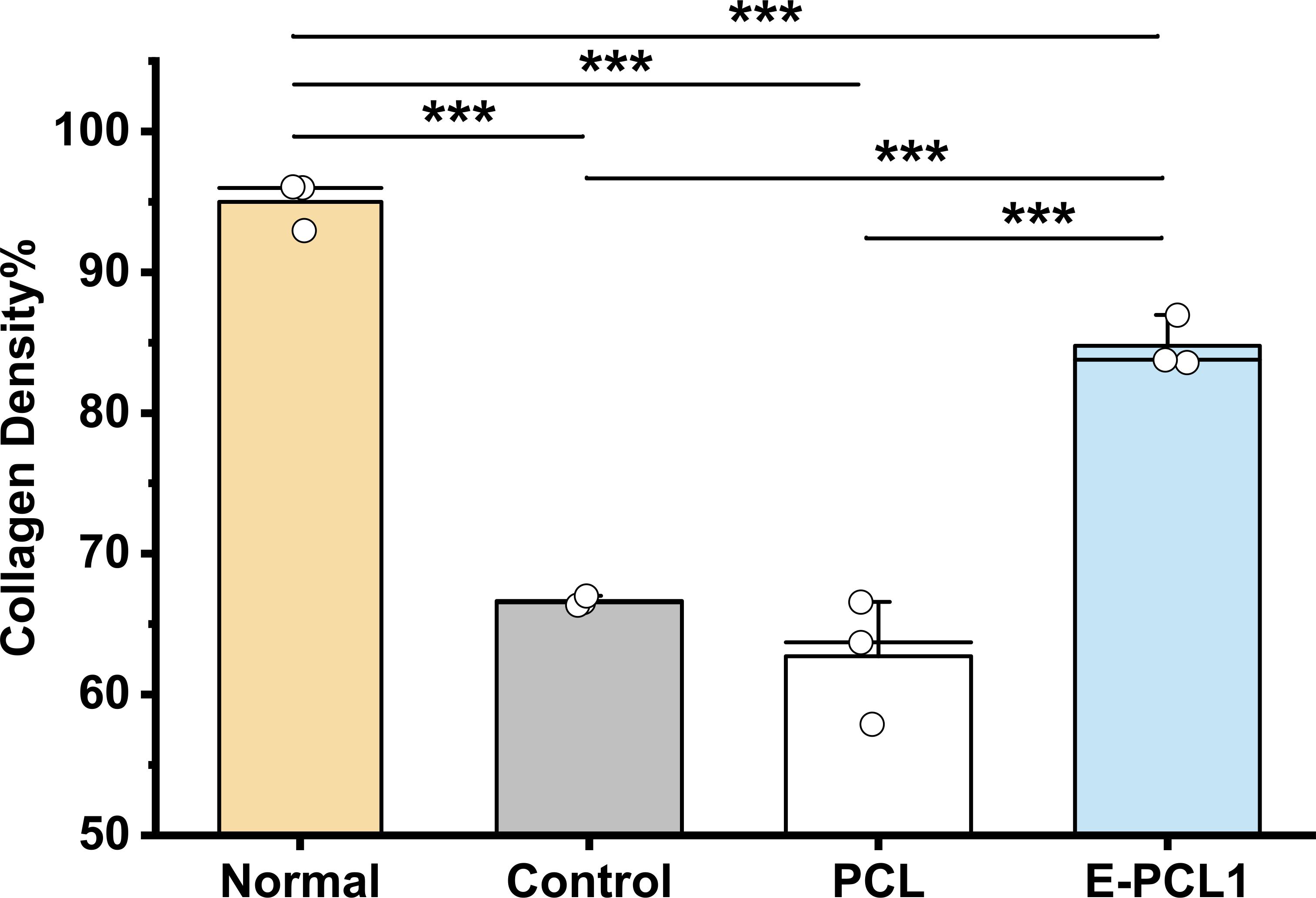


**Figure S25** Quantitative analysis of the collage density in normal, control, PCL, and E-PCL1 group (n = 3 biologically independent experiment).


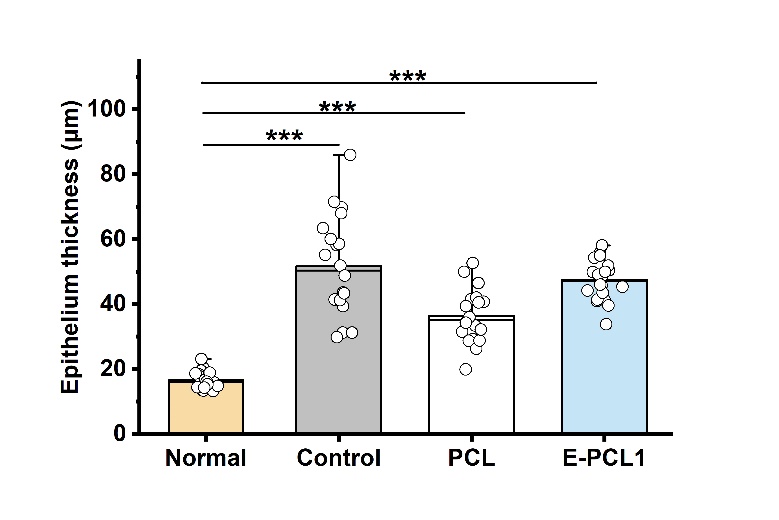


**Figure S26** Quantitative analysis of the epithelium thickness in normal, control, PCL, and E-PCL1 group (n = 3 biologically independent experiment).


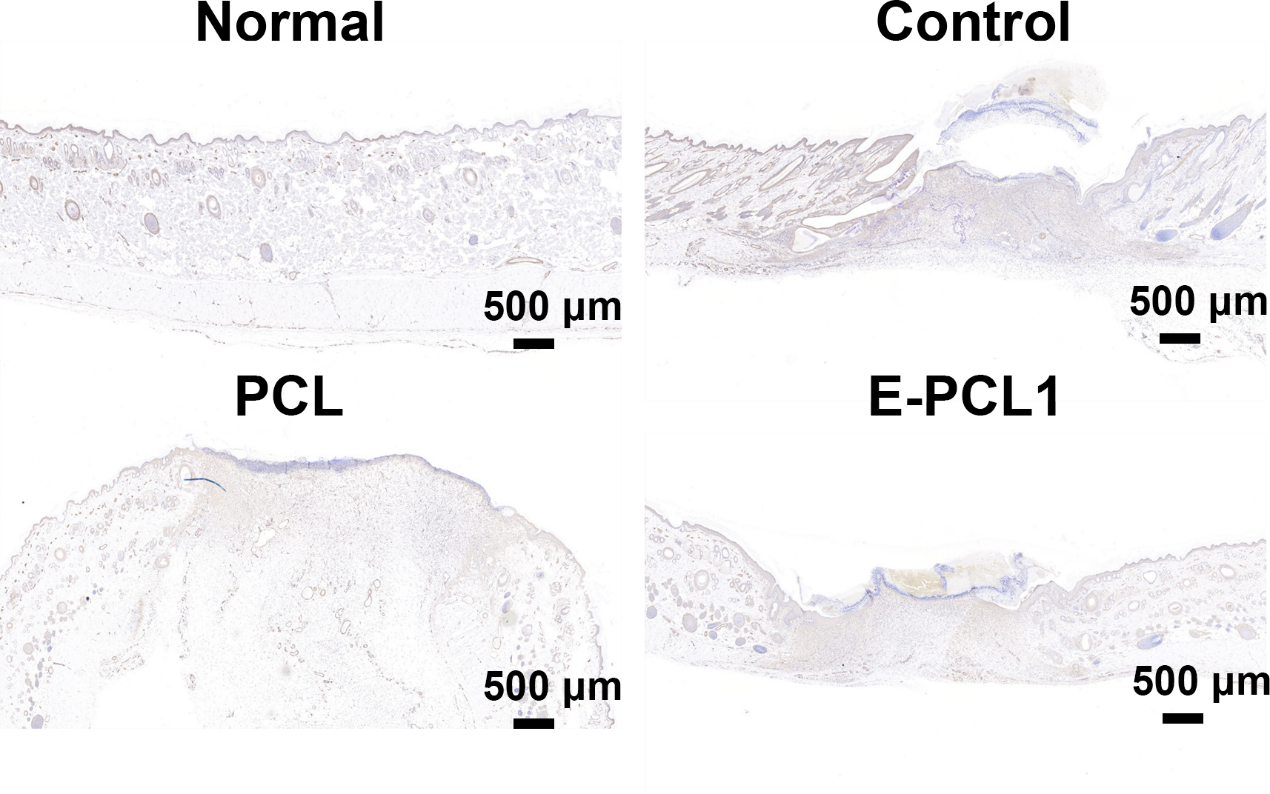


**Figure S27** Images of the α-SMA staining on day 7. (n = 3 biologically independent samples).


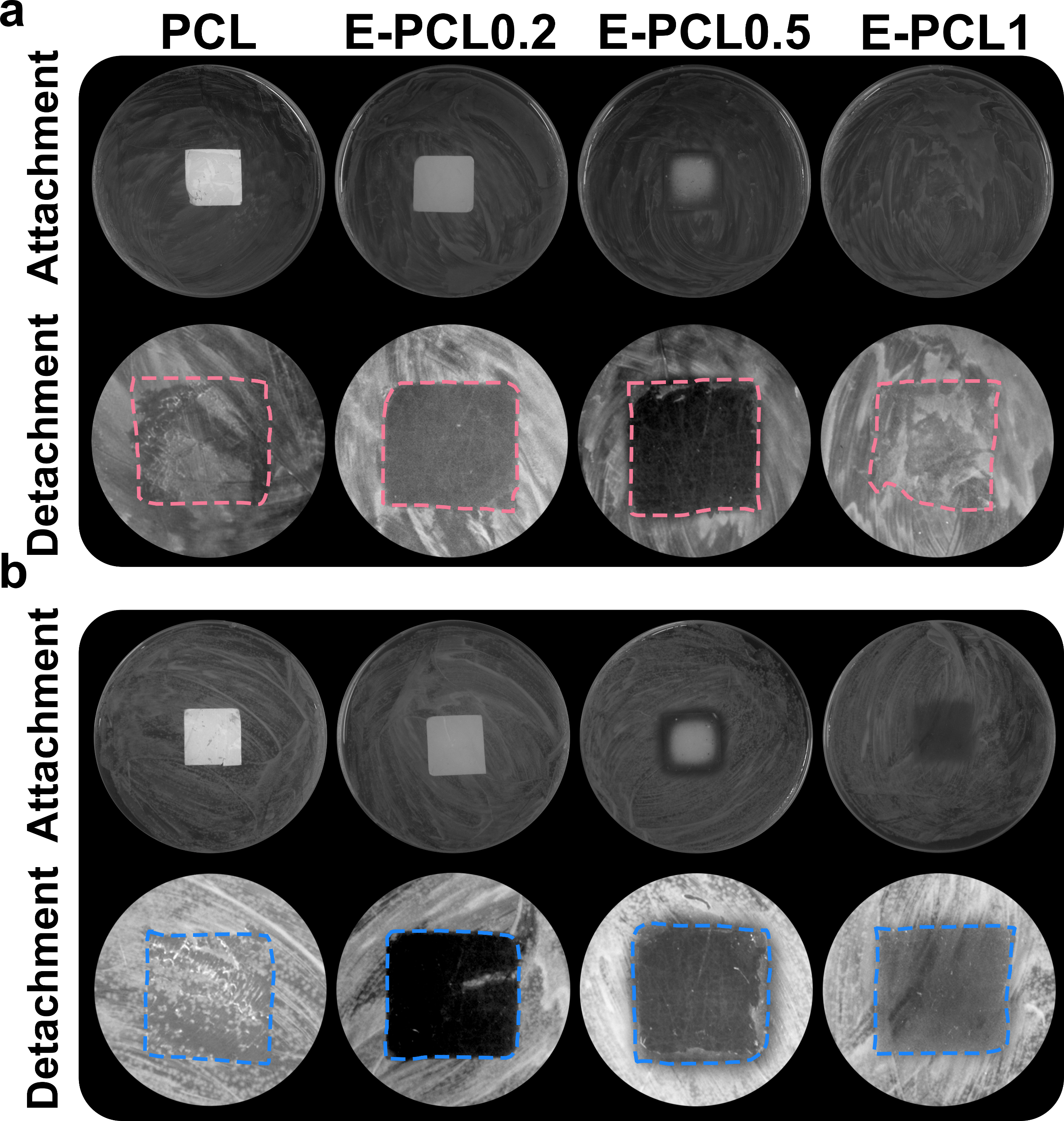


**Figure S28** The antibacterial results of PCL, E-PCL0.2, E-PCL0.5, and E-PCL1 against a) *Escherichia coli* and b) *Staphylococcus aureus*. The images of detachment are a close-up view, and the dashed box represents the original position of scaffolds.


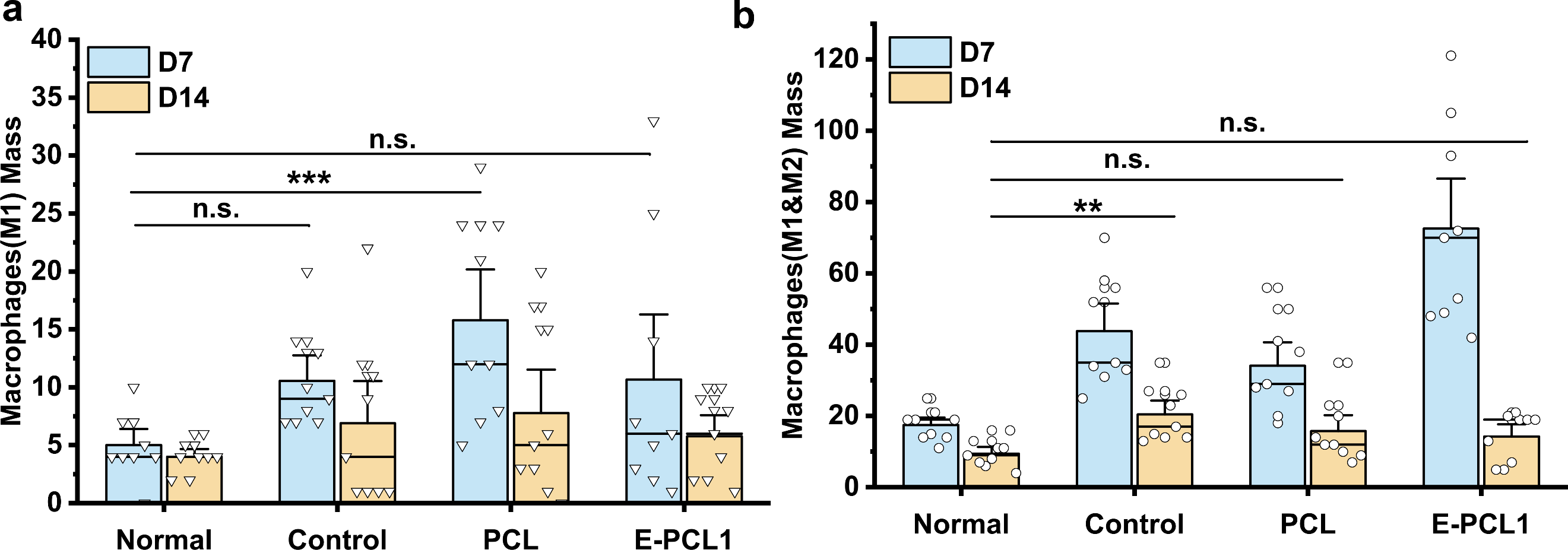


**Figure S29** Quantitative analysis of the macrophages (M1) and macrophages (M1&M2) on day 7 and 14, respectively (n = 3 biologically independent samples).


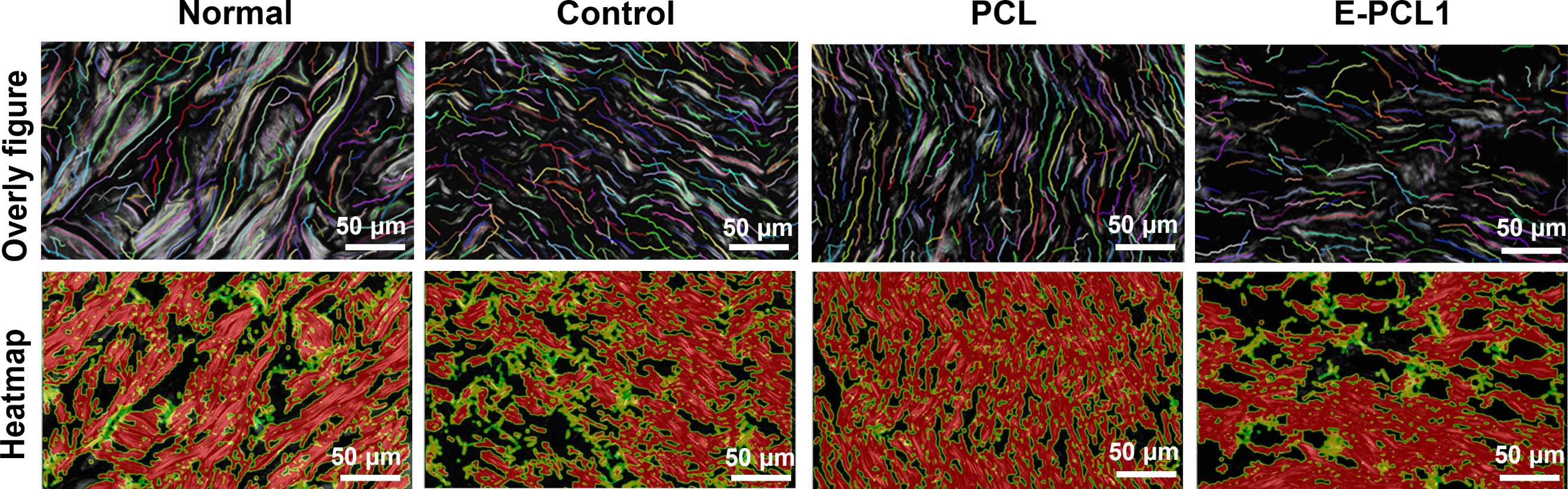


**Figure S30** Representative images of the Sirius red staining with software algorithms CurveAlign (https://loci.wisc.edu/curvealign/) on day 21.
